# Increased expression of *EHF* via gene amplification contributes to the activation of HER family signaling and associates with poor survival in gastric cancer

**DOI:** 10.1038/cddis.2016.346

**Published:** 2016-10-27

**Authors:** Jing Shi, Yiping Qu, Xinru Li, Fang Sui, Demao Yao, Qi Yang, Bingyin Shi, Meiju Ji, Peng Hou

**Affiliations:** 1 Department of Endocrinology, The First Affiliated Hospital of Xi'an Jiaotong University, Xi'an 710061, China; 2 Department of Surgery, The First Affiliated Hospital of Xi'an Jiaotong University, Xi'an 710061, China; 3 Key Laboratory for Tumor Precision Medicine of Shaanxi Province, The First Affiliated Hospital of Xi'an Jiaotong University, Xi'an 710061, China; 4 Center for Translational Medicine, The First Affiliated Hospital of Xi'an Jiaotong University, Xi'an 710061, China

## Abstract

The biological function of E26 transformation-specific (ETS) transcription factor EHF/ESE-3 in human cancers remains largely unknown, particularly gastric cancer. The aim of this study was to explore the role of EHF in tumorigenesis and its potential as a therapeutic target in gastric cancer. By using quantitative RT-PCR (qRT-PCR), immunohistochemistry (IHC) and fluorescence *in situ* hybridization (FISH) assays, we investigated the expression and copy number of *EHF* in a cohort of gastric cancers and control subjects. Specific *EHF* siRNAs was used to determine the biologic impacts and mechanisms of altered EHF expression *in vitro* and *in vivo*. Dual-luciferase reporter, chromatin immunoprecipitation (ChIP) and electrophoretic mobility shift assay (EMSA) assays were performed to identify its downstream targets. Our results demonstrated that *EHF* was significantly upregulated and frequently amplified in gastric cancer tissues as compared with control subjects. Moreover, *EHF* amplification was positively correlated with its overexpression and significantly associated with poor clinical outcomes of gastric cancer patients. We also found that EHF knockdown notably inhibited gastric cancer cell proliferation, colony formation, migration, invasion and tumorigenic potential in nude mice and induced cell cycle arrest and apoptosis. Importantly, we identified EHF as a new *HER2* transcription factor and the modulator of *HER3* and *HER4* in gastric cancer. Collectively, our findings suggest that *EHF* is a novel functional oncogene in gastric cancer by regulating the human epidermal growth factor receptor (HER) family of receptor tyrosine kinases and may represent a potential prognostic marker and therapeutic target for this cancer.

Gastric cancer is the fourth most common cancer and the second leading cause of cancer-related deaths in the world.^1, 2^ Given that most of patients are usually diagnosed at advanced stages in developing countries and the outcomes are still poor, the early diagnosis is thus extremely important for good prognosis.^3, 4^ Like other cancers, multiple genetic and epigenetic alterations have a critical role in the pathogenesis of gastric cancer. Therefore, identification of prognostic and/or therapeutic targets may improve early diagnosis and treatment efficacy for this disease.

The E26 transformation-specific (ETS) transcription factor family is one of the largest transcription factor families, which can be structurally categorized into 11 subfamilies including ETS, ERG, ELG, ELF, ERF, TEL, PEA3, SPI, TCF, PDEF and ESE.^5, 6, 7, 8^ All ETS factors are characterized by the ETS domain, which is a highly conserved DNA-binding domain comprising ~85 amino acids that form the winged helix-turn-helix DNA-binding motif, and recognizes the core consensus DNA sequence 5′-GGA(A/T)-3′ (ETS-binding site, EBS).^9, 10^ Some of them have been found to alter expression patterns involving diverse mechanisms, such as gene fusion and chromosomal rearrangements,^11, 12^ amplification and/or overexpression,^13, 14^ point mutations.^15^ Thus, the ETS factors have a key role in various diseases, including cancers.^8, 16, 17^

EHF/ESE-3 is new member of the ETS transcription factors. Like ESE-1 and ESE-2, it is exclusively expressed in a subset of epithelial cells.^18^ The previous studies have showed that altered expression of EHF may affect the normal process of epithelial cell differentiation and contribute to cell transformation.^18, 19, 20^ Moreover, EHF may regulate epithelial growth and differentiation and have an important role in oncogenesis of epithelium-derived tumors.^18, 21^ *EHF* has been demonstrated to function as a potential tumor suppressor gene in prostate cancer and esophageal squamous cell carcinoma (ESCC),^19, 22, 23^ and be frequently downregulated by promoter methylation.^22^ On the other hand, it is overexpressed in ovarian and mammary cancers^24, 25, 26^ and may be a predictive marker for poor survival in ovarian cancer.^24, 25^ However, the roles of EHF in gastric tumorigenesis are not fully understood.

In this study, we found frequent *EHF* overexpression and genomic amplification in gastric cancers, and demonstrated *EHF* amplification was one of the major mechanisms for its overexpression. Importantly, we for the first time revealed a close association of *EHF* amplification with poor patient survival. EHF downregulation significantly reduced *in vitro* and *in vivo* oncogenic potential of gastric cancer cells by regulating HER family of receptor tyrosine kinases.

## Results

### *EHF* is highly expressed and amplified in gastric cancers

**Figure 1:**
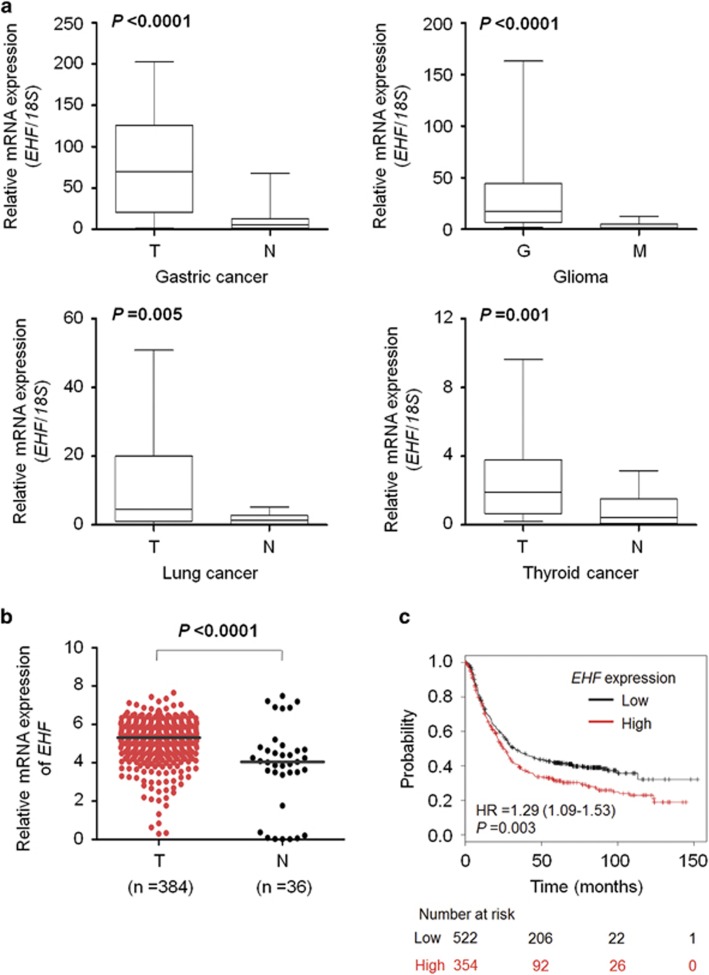
*EHF* is highly expressed in multiple cancers. (**a**) qRT-PCR assay was performed to evaluate mRNA expression of *EHF* in primary gastric cancers (T) and their matched non-cancerous gastric tissues (N; *n*=30), gliomas (G; *n*=18) and meningiomas (M; *n*=19), primary lung cancers (T) and their matched non-cancerous tissues (N; *n*=18), as well as primary thyroid cancers (T) and their matched non-cancerous tissues (N; *n*=20), respectively. *EHF* expression was normalized with *18S* rRNA levels. (**b**) High expression of *EHF* in gastric cancers (T) compared with normal gastric tissues (N) in TCGA data set. Horizontal lines indicate the median. (**c**) Significant correlation between *EHF* expression and the survival of gastric cancer patients in the Kaplan–Meier Plotter database

We first examined *EHF* expression in a cohort of tumor tissues including gastric cancers, gliomas, lung cancers and thyroid cancers and non-cancerous tissues. As shown in Figure 1a and Supplementary Figure 1, compared with matched non-cancerous gastric tissues, *EHF* was significantly upregulated in gastric cancer tissues at both mRNA and protein levels. Similarly, we found that EHF expression was higher in gastric cancer cell lines than gastric mucosal epithelial cell line GES-1 (Supplementary Figure 2a and b). Moreover, *EHF* was also significantly upregulated in gliomas (*P*<0.0001), lung cancers (*P*=0.005) and thyroid cancers (*P*=0.001) compared with control subjects. We also investigated *EHF* expression in a total of 384 stomach adenocarcinomas using TCGA data set from the Cancer Browser database.^27^ As expected, *EHF* expression in tumor tissues was significantly higher than that in normal controls (*P*<0.0001; Figure 1b). In addition, we found that *EHF* expression was positively correlated with poor survival in gastric cancer patients (data from the Kaplan–Meier Plotter; *P*=0.003; Figure 1c).

**Figure 2:**
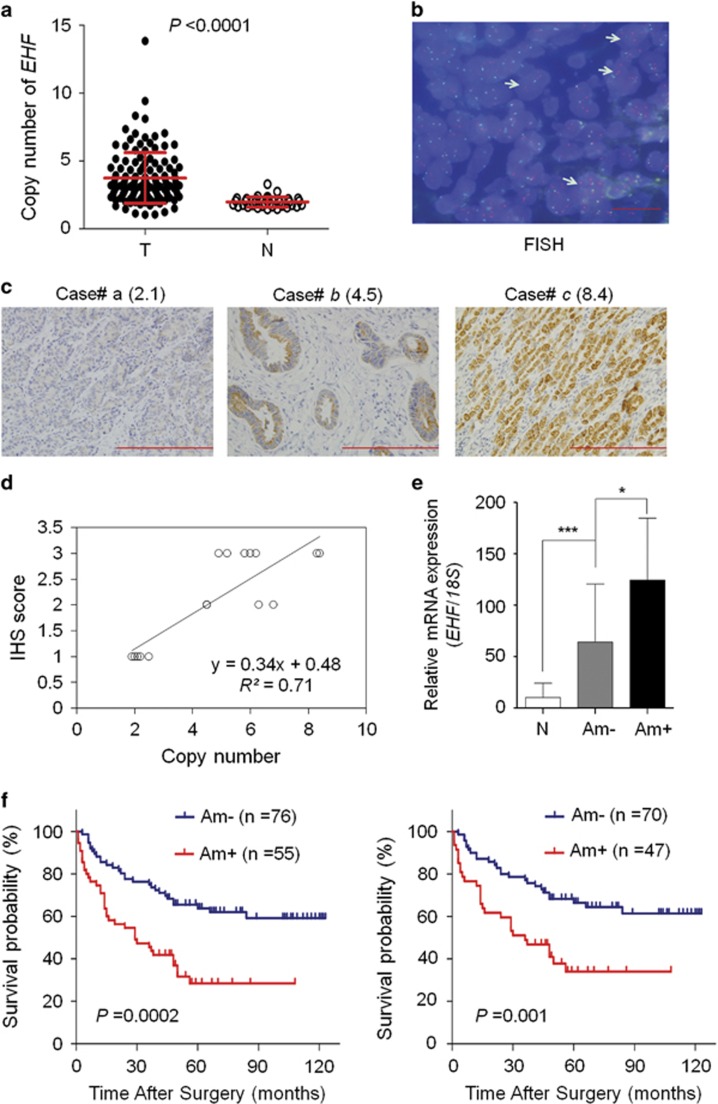
*EHF* is frequently amplified in gastric cancers. (**a**) Real-time quantitative PCR was performed to analyze *EHF* copy number in a cohort of gastric cancers (T) and control subjects (N). Horizontal lines indicate mean±S.E. (**b**) Bicolor FISH analysis demonstrates *EHF* amplification (red signals) in primary gastric cancer tissues by using EHF DNA probe, and reference centromeric probe on chromosome 11 (CEN11) was shown in green. Arrows indicate the cells with *EHF* amplification. Magnification for each set: × 1000. Scale bars, 200 *μ*m. (**c**) Increasing extent of specific staining (brown color) was associated with increasing *EHF* copy number (number inside brackets). Shown are representative images of IHC on gastric cancer histologic slides using anti-EHF antibody. Magnification for each set: × 400. Scale bars, 200 *μ*m. (**d**) Linear regression analysis was performed to evaluate the relationship between EHF immunohistostaining score and *EHF* copy number on 16 randomly selected gastric cancer cases (*R^2^*=0.71). (**e**) *EHF* mRNA expression was analyzed by using qRT-PCR assay in primary gastric cancers (*n*=30) grouping with *EHF* amplification and matched normal tissues (N). *18S* rRNA was used as a normalized control. Data are presented as mean±S.E. (**f**) Kaplan–Meier survival curves were used to assess patient survival. The patients with *EHF* amplification (Am+) had significantly shorter survival times than those without *EHF* amplification(Am−) (left panel). When the patients with residual cancers were excluded, the patients with *EHF* amplification still had significantly poor survival compared with those without *EHF* amplification (right panel). Statistically significant differences were indicated: *P<0.05; ***P<0.001

Given that genomic amplification is one of the major causes of oncogene overexpression in human cancers including gastric cancer,^28^ we analyzed the copy number of *EHF* in 131 gastric cancers and 37 control subjects by using qRT-PCR assay. *EHF* copy number corresponding to each individual case was presented in Figure 2a. Further analysis showed that *EHF* copy number in gastric cancers was significantly higher than that in control subjects (*P*<0.0001). We also analyzed *EHF* copy number in cell lines. The results showed that *EHF* copy number was higher in BGC823 and SGC7901 cells than GES-1, MGC803 and AGS cells (Supplementary Figure 2c). With a gene copy number of 4 or more defined as gene amplification, *EHF* amplification was detected in 55 of 131 (41.98%) gastric cancers, but not in control subjects. This finding was supported by fluorescence *in situ* hybridization (FISH) analysis (Figure 2b).

To test the relationship between of *EHF* copy number and its expression, we randomly selected 16 gastric cancer cases with different *EHF* copies and did immunohistochemistry (IHC) for EHF. Increased staining of EHF was seen with increased *EHF* copies (Figure 2c). Linear regression analysis on the 16 cases revealed a positive correlation between the IHS score and copies of *EHF* (*R*^2^=0.71; Figure 2d). Similarly, our data showed that there was a significantly positive relationship between mRNA expression of *EHF* and its genomic amplification (*P*=0.017; Figure 2e). However, *EHF* expression was still higher in the cases without *EHF* amplification than matched normal gastric tissues (*P*<0.001), suggesting the existence of other possible mechanisms leading to its overexpression.

### *EHF* amplification is associated with poor clinical outcomes in gastric cancer

We investigated the association of *EHF* amplification with clinicopathologic features and clinical outcomes in a cohort of gastric cancers. As shown in Supplementary Table 1, *EHF* amplification was significantly positively associated with differentiation (*P*=0.049), lymph node metastasis (*P*=0.029) and survival status (*P*=0.001). We further conducted a multiple multivariable logistic regression (Supplementary Table 2). As exppected, after adjustment, *EHF* amplification was still closely correlated with survival status (OR=3.748, 95% CI=1.525–9.214; *P*=0.004).

Next, Kaplan–Meier method was used to determine the effect of *EHF* amplification on patient survival. The data showed that the patients with *EHF* amplification had significantly shorter median survival times than those without *EHF* amplification (32.2 months *versus* 62.0 months; *P*=0.0002; Figure 2f, left panel). Accumulated evidences have demonstrated that residual tumor after surgery is an independent risk factor for gastric cancer patients.^28^ Thus, we excluded the cases with residual tumor to evaluate the effect of *EHF* amplification on patient survival. The results showed that *EHF* amplification still significantly shortened patient survival times (34.4 months *versus* 63.8 months, *P*=0.001; Figure 2f, right panel). Moreover, univariate cox regression analysis indicated that *EHF* amplification was significantly associated with poor survival with a hazard ratio (HR) of 2.593 (95% CI=1.583–4.249; *P*=0.0002; Table 1). Multivariate cox regression analysis further demonstrated that *EHF* amplification might be an independent prognostic factor in gastric cancer (HR=2.426; 95% CI=1.474–3.991; *P*<0.0001; Table 1).

### EHF promotes gastric cancer cell growth

**Figure 3:**
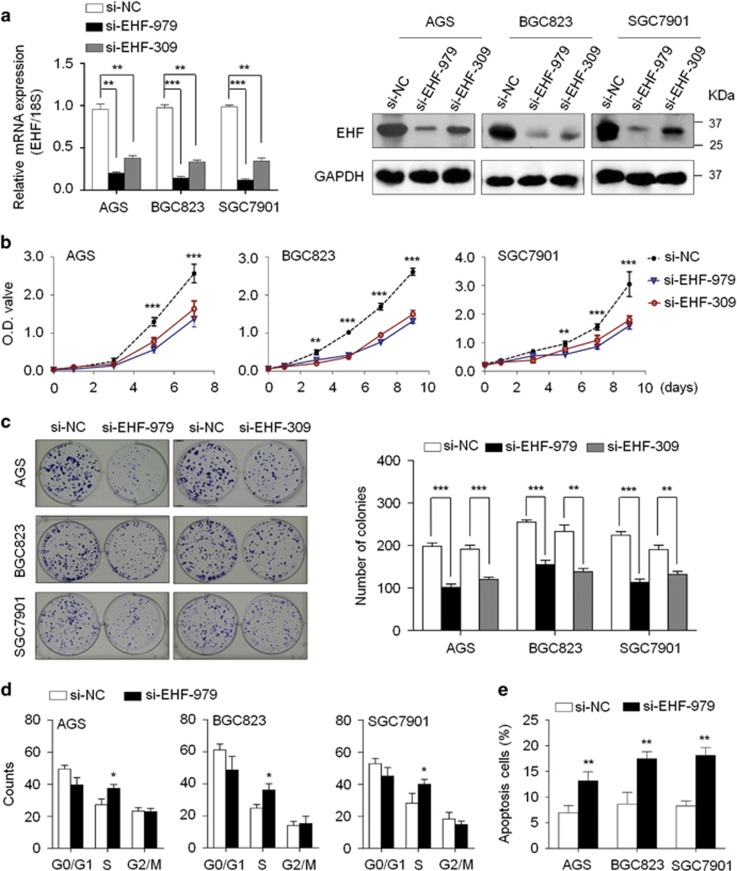
EHF knockdown inhibits cell growth and induces cell cycle arrest and apoptosis in gastric cancer cells. (**a**) Knockdown of *EHF* mRNA (left panels) and protein (right panels) by using two different siRNAs (si-EHF-979 and si-EHF-309) in gastric cancer cell lines was evidenced by qRT-PCR and western blot assays, respectively. *18S* rRNA was used as a normalized control for qRT-PCR assay. GAPDH was used as loading control in western blot analysis. (**b**) EHF knockdown significantly inhibited cell proliferation in gastric cancer cells. (**c**) Left panel shows the representative images of colony formation in cells transfected with si-EHF or si-NC. Quantitative analysis of colony numbers is shown in right panel. (**d**) The indicated cells were transiently transfected with si-EHF-979 or si-NC. DNA content was measured by flow cytometry to determine cell cycle fractions. (**e**) Apoptotic cells including early and late apoptotic cells were measured 72 h after transfection by flow cytometry analysis of Annexin V-FITC/PI double-labelled cells. The data were presented as mean±S.E. of values from three independent experiments. Statistically significant differences were indicated: *P<0.05; **P<0.01; ***P<0.001

To elucidate the role of EHF in gastric carcinogenesis, we tested the growth-suppressive effect by knocking down *EHF* expression in gastric cancer cell lines AGS, BGC823 and SGC7901 using siRNA approach. EHF knockdown by two different *EHF* siRNAs (si-EHF-979 and si-EHF-309) was confirmed by qRT-PCR and western blot assays (Figure 3a). These two specific siRNAs significantly inhibited cell proliferation (Figure 3b) and colony-forming ability in monolayer culture (Figure 3c) compared with control siRNA (si-NC). Conversely, ectopic expression of *EHF* in GES-1 and MGC803 cells significantly promoted cell proliferation compared with empty vector (Supplementary Figure 3). Altogether, these results suggest the growth-promoting role of EHF in gastric tumorigenesis.

### EHF knockdown induces gastric cancer cell cycle arrest and apoptosis

We next evaluated the effect of EHF knockdown on cell cycle distributions and apoptosis in gastric cancer cells. The results showed that cell cycle was arrested at the S phase in si-EHF-979 transfected cells compared with si-NC transfected cells (Figure 3d). This effect could be reversed by exogenous overexpressing EHF in these three cell lines (Supplementary Figure 4). In addition, si-EHF-979 transfection caused an increase in the numbers of apoptotic cells compared with control cells (Figure 3e).

### EHF knockdown inhibits gastric cancer cell growth in nude mice

**Figure 4:**
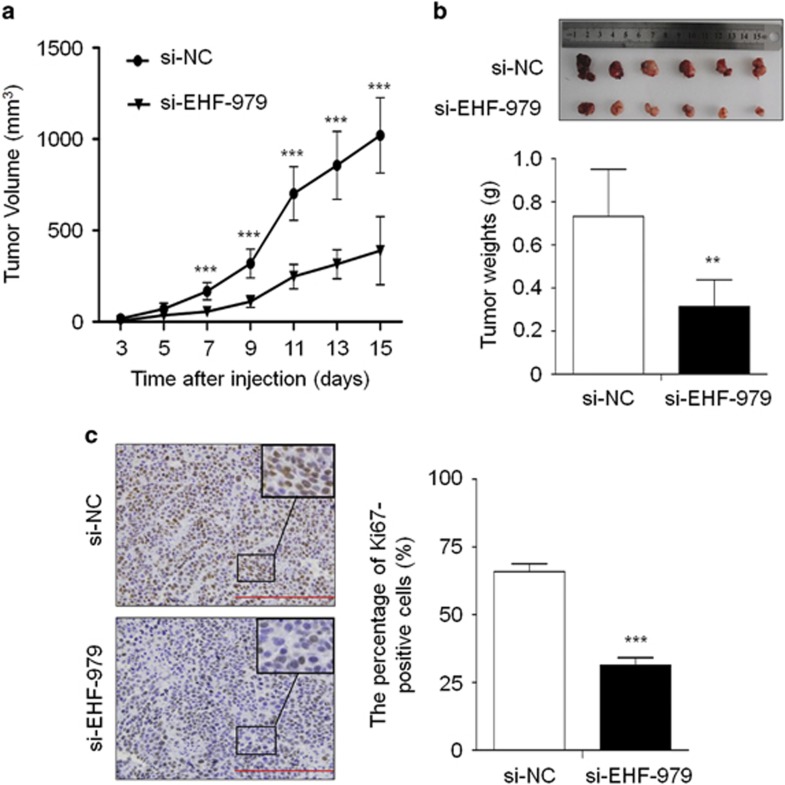
EHF knockdown inhibits xenograft tumor growth. (**a**) Subcutaneous tumor growth curve of si-EHF-979 transfected BGC823 cells in nude mice was compared with si-NC transfected cells. The si-EHF-979 group showed a retarded tumor growth compared to the si-NC group. Data are shown as mean±S.D. (*n*=6 per group). (**b**) A representative picture for tumor growth of cells transfected with the indicated siRNA in nude mice (upper panel). Histogram represents mean of tumor weight from the si-EHF-979 and si-NC groups (lower panel). Data are shown as mean±S.D. (*n*=6 per group). (**c**) Shown is representative Ki-67 staining of xenograft tumors from the si-EHF-979 and si-NC groups(left panels). Histogram represents mean±S.E. of the percentage of Ki-67-positive cells from five microscopic fields in each group (right panel). Magnification for each set: × 400. Scale bars, 200 *μ*m. Statistically significant differences were indicated: **P<0.01; ***P<0.001

We assessed the effect of EHF knockdown on the growth of xenograft tumors in nude mice. As shown in Figure 4a, tumors induced by si-EHF-979 transfected BGC823 cells showed significantly longer latency and smaller mean tumor volume than tumors induced by control cells. At the end of experiments, tumors were isolated and weighed. The mean weight of si-EHF-979 transfected cell-derived tumors was significantly less compared with control tumors (*P*=0.002; Figure 4b). Moreover, we analyzed the expression levels of EHF in xenogaft tumors by using qRT-PCR and western blot assays. As shown in Supplementary Figure 5, EHF expression was significantly decreased in si-EHF-979 group compared with si-NC groups at both mRNA and protein levels. These data suggest that transient transfection of si-EHF-979 may knockdown EHF expression and inhibit the growth of xenogaft tumors within 15 days after injection, although there may be off-target effect. To assess the proliferation index in the xenograft tumor, tumor sections were stained with anti-Ki-67 antibody. The percentage of Ki-67-positive cells was significantly decreased in si-EHF-979 transfected cell-derived tumors compared with control tumors (*P*<0.001; Figure 4c). These observations further support that *EHF* is a functional oncogene in gastric cancer cells.

### EHF enhances gastric cancer cell migration and invasion

**Figure 5:**
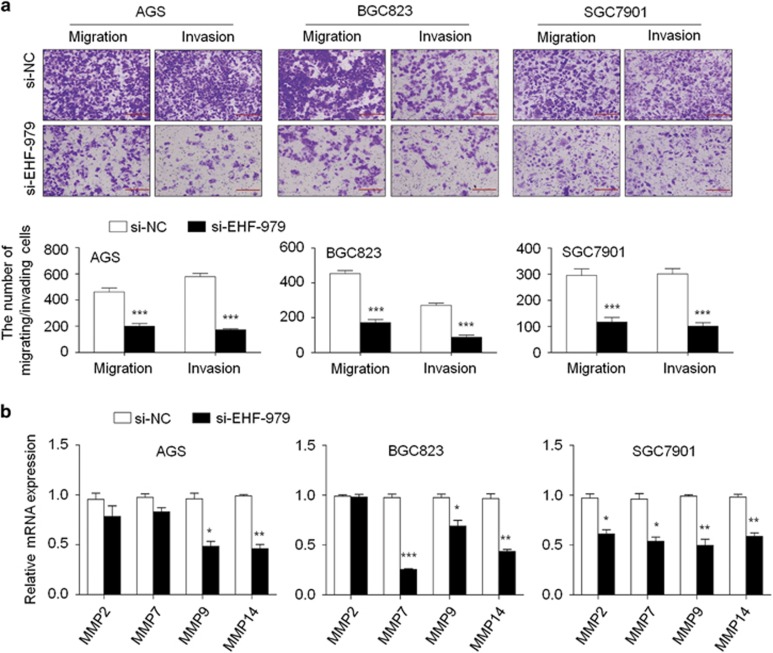
EHF knockdown inhibits gastric cancer cell migration and invasion. (**a**) The representative images of migrated/invaded cells (upper panels). Histograms, corresponding to upper panels, show means±S.E. of cell numbers from three independent assays (lower panels). Magnification for each set: × 200. Scale bars, 50 *μ*m. (**b**) qRT-PCR was performed to test the effect of EHF depletion on the expression of metastasis-related genes *MMP2*, *7*, *9* and *14* in gastric cancer cells. Expression levels of these genes were normalized with *18S* rRNA levels. Data were presented as mean±S.E. *P<0.05;**P<0.01; ***P<0.001

We also attempted to test the effects of EHF on gastric cancer cell migratory and invasive ability. There were a significantly lower number of migrated/invaded cells in si-EHF-979 transfected cells than si-NC transfected cells (Figure 5a). Conversely, ectopic expression of EHF in MGC803 cells significantly enhanced the migration and invasive potential of cancer cells (Supplementary Figure 6). Given an important role of matrix metalloproteinases (MMPs) in cell invasiveness,^29^ we next tested the effect of EHF on the expression of *MMP-2*, *-7*, *-9* and *-14*. EHF knockdown significantly inhibited the expression of these genes in at least one cell lines (Figure 5b), whereas overexpressing EHF in MGC803 cells increased the expression of these genes (Supplementary Figure 7a). These data indicate that metastasis-associated phenotypes may be link to the regulation of MMPs by EHF in gastric cancer.

**Figure 6:**
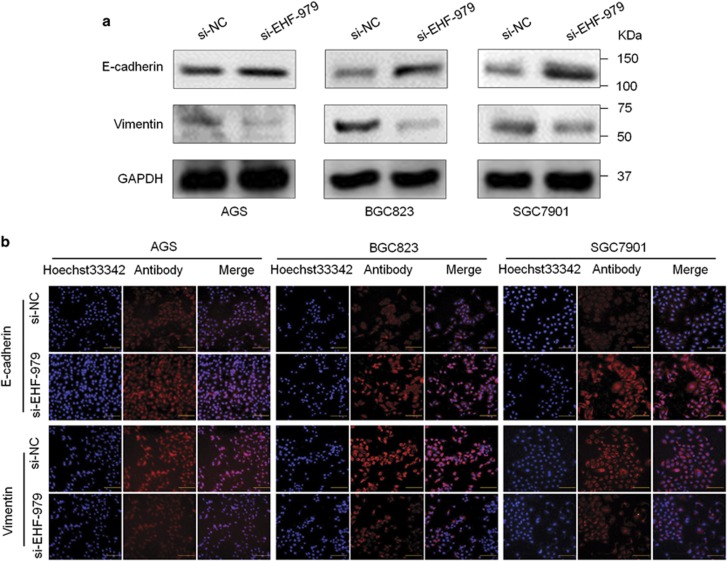
EHF knockdown inhibits EMT process in gastric cancer cells. (**a**) The expression of E-cadherin and Vimentin was determined in the indicated cells by western blot analysis. GAPDH was used as loading control. (**b**) Immunofluorescence staining was then used to assess the expression of E-cadherin and Vimentin proteins in cells transfected with si-EHF-979 or si-NC. Red color represents target protein fluorescence and blue color represents Hoechst33342 staining for nuclei. Magnification for each set: × 400. Scale bars, 100 *μ*m

We further tested the effect of EHF on the process of epithelial–mesenchymal transition (EMT), which is one of the critical steps during tumor metastasis including gastric cancer.^30^ As shown in Figure 6a, knocking down EHF expression in AGS, BGC823 and SGC7901 cells increased E-cadherin expression, and decreased Vimentin expression. Conversely, overexpressing EHF in MGC803 cells decreased E-cadherin expression and increased Vimentin expression (Supplementary Figure 7b). These findings were also supported by immunofluorescence (IF) assay in Figure 6b. Collectively, these results suggest that EHF contributes to gastric cancer cell metastasis via promoting EMT process.

### EHF modulates the activities of the HER family of receptor tyrosine kinases in gastric cancer cells

**Figure 7:**
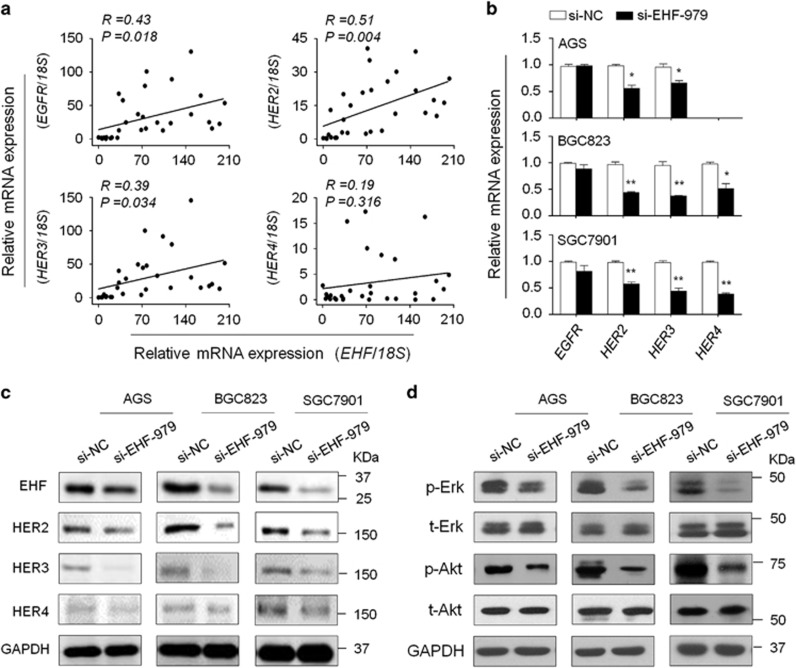
EHF regulates the expression of HER receptors and the activities of their downstream signaling pathways in gastric cancer cells. (**a**) qRT-PCR assay was used to evaluate mRNA expression of *EHF* and HER receptors in primary gastric cancers (*n*=30). Linear regression analysis was performed to assess the correlations between them. *18S* rRNA was used as a normalized control. (**b**) qRT-PCR assay was performed to investigate the effect of EHF knockdown on the expression of HER receptors. Expression levels of these genes were normalized with *18S* rRNA levels. Data were presented as mean±S.E. (**c**) The effect of EHF depletion on the expression of HER2-4 was determined in the indicated cells by western blot analysis. GAPDH was used as loading control. (**d**) Cells transfected with si-EHF-979 or si-NC were lysed and lysates were subjected to western blot analysis. The antibodies against phospho-Erk (p-Erk), total Erk (t-Erk), phospho-Akt (p-Akt) and total Akt (t-Akt) were used to determine the effect of EHF knockdown on the activities of the MAPK/Erk and PI3K/Akt cascades. GAPDH was used as a loading control

The HER family includes four members: EGFR/HER1, HER2, HER3 and HER4.^31^ Alterations in HER family play a critical role in the progression and survival of many cancers including gastric cancer.^32^ In addition, we found the core GGAA/T motif (EBS) in the promoters of *EGFR*, *HER2*, *HER3* and *HER4* by analyzing their promoter sequences using MatInspector online software (http://www.genomatix.de/online_help/help_matinspector/matinspector_help). Thus, we attempted to determine whether oncogenic role of EHF in gastric cancer is associated with the activation of HER family. As shown in Figure 7a, *EHF* expression was significantly positively correlated with the expression of *EGFR* (*R*=0.43; *P*=0.018), *HER2* (*R*=0.51; *P*=0.004) and *HER3* (*R*=0.39; *P*=0.034) in gastric cancers. Similarly, we found a positive association of *EHF* expression with the expression of *HER2* (*R*=0.29; *P*<0.0001) and *HER3* (*R*=0.36; *P*<0.0001) by using TCGA dataset from the Cancer Browser database (Supplementary Figure 8). In addition, EHF knockdown dramatically decreased the expression of HERs in the indicated cell lines compared with the controls, particularly HER2 and HER3 (Figure 7b and c). On the other hand, overexpressing EHF in MGC803 cells upregulated the expression of HER2-4 (Supplementary Figure 9). Interestingly, we found that the expression of *BMP1*, *BMP4* and *c-Met* was decreased by EHF knockdown in at least two cell lines compared with the controls (Supplementary Figure 10). As expected, EBS could also be found in their promoters.

Increasing evidences have demonstrated that overexpression of HER family members leads to the activation of downstream pathways including the MAPK/Erk and PI3K/Akt pathways.^33^ As expected, EHF knockdown significantly inhibited the activities of both pathways in gastric cancer cells, characterized by reduced phosphorylation of Erk (p-Erk) and Akt (p-Akt) (Figure 7d). This was supported by a very recent study that the levels of p-Erk and p-Akt were inhibited by EHF knockdown in ovarian cancer cells.^25^

**Figure 8:**
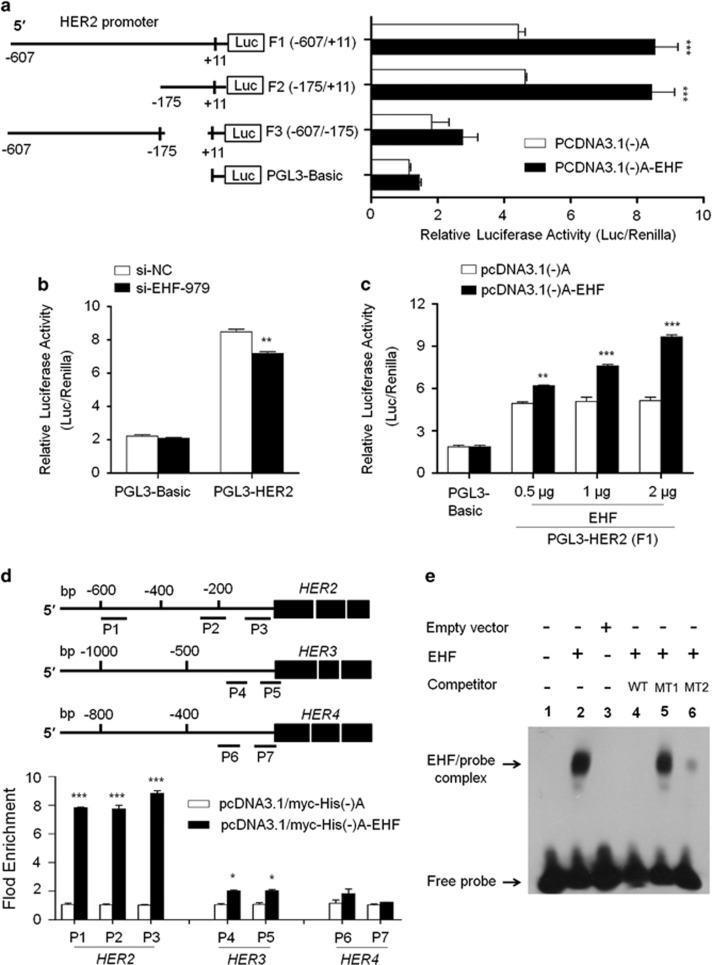
EHF is identified as a new HER2 transcription factor and the modulator of HER3 and HER4 in gastric cancer. (**a**) BGC823 cells were transiently transfected with pGL3-Basic or luciferase reporter constructs containing various lengths of the promoter region of *HER2* gene, as indicated (F1: −607/+11; F2: −175/+11; F3: −607/−175) (left panels). Cotransfection with empty vector was used as a control. The ratio of the Luc/Renilla activity is shown as means±S.E. of three independent assays (right panels). (**b**) The luciferase reporter gene assay was performed to evaluate the effect of *EHF* knockdown on promoter activity of *HER2* in BGC823 cells. The ratio of the Luc/Renilla activity is shown as means±S.E. of three independent assays. (**c**) HEK293T cells were cotransfected pGL3-HER2-Luc-F1 and various amounts of pcDNA3.1(-)A-EHF or empty vector, respectively. Promoter activities of *HER2* were measured by luciferase reporter gene assays. All the ratio of the Luc/Renilla activity is shown as means±S.E. of three independent assays. (**d**) Putative promoter regions of *HER2* (−607/+11), *HER3* (−997/+440) and *HER4* (−697/+306) were inserted into the pGL3-Basic to construct the luciferase reporter plasmid pGL3-HER2-Luc, pGL3-HER3-Luc and pGL3-HER4-Luc (upper panels). P1-P7 represent the regions analyzed by ChIP assays for *HER2*, *HER3* and *HER4*, respectively. BGC823 cells were transiently transfected with pcDNA3.1/myc-His(-)A-EHF or empty vector, and were subjected to ChIP-qRT-PCR assays using anti-Myc tag antibody. Flod enrichment was shown as means±S.E. of three independent assays (lower panels). (**e**) EMSA assay was performed to confirm the interaction between EHF and *HER2* promoter. Shown are specific DNA-binding of *in vitro* translated EHF protein to an oligonucleotide sequence (SH2) containing ETS responsive element (GAGGAA) from the *HER2* promoter. Unlabeled mutated probes contain specific mutations in the GGAA ETS core or flanking nucleotides of core sequence, as indicated by MT1 and MT2. Unlabeled wild-type (WT) and mutated (MT1 or MT2) competitor probes were added at 100-fold molar excess. Statistically significant differences were indicated: *P<0.05; **P<0.01; ***P<0.001

To examine whether EHF was indeed involved in directly regulating promoter activities of *HER2*-*4*, we cloned their promoters into a pGL3-Basic luciferase plasmid to construct luciferase reporter plasmids including pGL3-HER2-Luc (−607/+11), pGL3-HER3-Luc (−997/+440) and pGL3-HER4-Luc (−697/+306). The results showed that ectopic expression of EHF was able to significantly increase promoter activity of *HER2*, but not *HER3* and *HER4*, in BGC823 cells (Figure 8a and Supplementary Figure 11a). Next, to further identify *HER2* promoter core region, three different lengths of *HER2* promoter region (F1: −607/+11; F2: −175/+11; F3: −607/−175 bp) were inserted into the pGL3-Basic luciferase plasmid, and co-transfected into BGC823 cell with pcDNA3.1(-)A-EHF or empty vector, respectively. The results showed that pGL3-HER2-Luc-F1 and -F2 exhibited strong luciferase activity, but not pGL3-HER2-Luc-F3, compared with pGL3-Luc-Basic (Figure 8a). On the other hand, EHF knockdown significantly decreased the promoter activity of *HER2* (*P*<0.01) (Figure 8b), but not *HER3* (Supplementary Figure 11b), in BGC823 cells. In addition, we found that the promoter activity of *HER2* in HEK293T cells was increased with the increased amounts of EHF-expressing plasmid (Figure 8c). Taken together, these observations suggest that EHF may be a potential transcription factor of *HER2*, and the regulation site of EHF is located at −175/+11 of *HER2* gene.

Next, we attempted to explore whether the activity of *HER2* was regulated by EHF through directly binding to its promoter. Thus, the chromatin immunoprecipitation (ChIP) assay was performed in BGC823 cells transfected with pcDNA3.1/myc-His(-)A-EHF and empty vector using anti-Myc tag antibody, followed by qRT-PCR targeting their promoter regions. As expected, EHF strongly bound to *HER2* promoter and weakly bound to *HER3* promoter, but not *HER4* in BGC823 cells (Figure 8d). Three different fragments within *HER2* promoter (P1: −604/−484; P2: −274/−155; P3: −147/-37) were all enriched by 8.14-fold on average in pcDNA3.1/myc-His(-)A-EHF-transfected cells compared with vector-transfected cells (*P*<0.001; Figure 8d). To be consistent with the dual luciferase findings, the ChIP assays further support *HER2* as a target of EHF.

**Figure 9:**
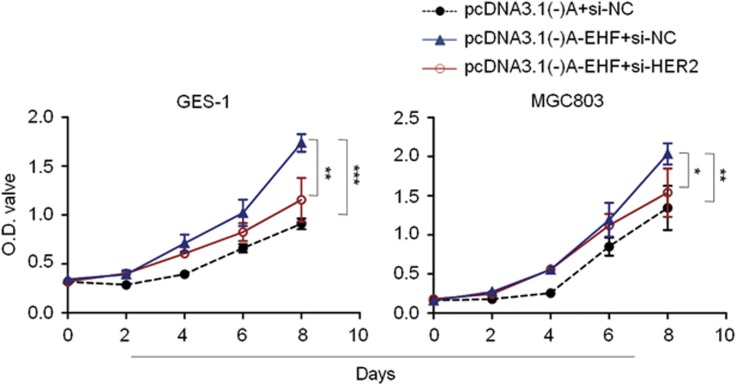
HER2 depletion attenuates proliferation-promoting effect of EHF in gastric cancer cells. Inhibitory effect of HER2 depletion on cell proliferation in GES-1 and MGC803 cells overexpressing EHF were evaluated by MTT assay. The data were presented as mean±S.E. Statistically significant differences were indicated: *P<0.05; **P<0.01; ***P<0.001

To test whether EHF directly interacts with *HER2* promoter, an oligonucleotide sequence (SH2) containing the putative Ets binding site (EBS; GAGGAA) from *HER2* promoter was used to assess DNA-binding and transactivation by *in vitro*-translated full-length EHF protein using EMSA assay. This oligonucleotide sequence has previously been demonstrated to be responsive to another ETS factor ESE1/ESX.^34^ As shown in Figure 8e, full-length EHF protein exhibited high-affinity, sequence-specific binding to SH2. Moreover, unlabeled specific competitor probes (WT) completely competed with SH2 for EHF binding. As seen with other ETS factors, unlabeled competitor probes (MT1; 100 fold) with mutations in the GGAA ETS core of SH2 failed to compete against SH2 for EHF binding, whereas those (MT2; 100 fold) with mutations in flanking nucleotides of core sequence were relatively effective at competing for EHF binding. To determine the role of HER2 in growth-promoting effect of EHF on gastric cancer cells, we knocked down HER2 expression in GES-1 and MGC803 cells overexpressing EHF. The results showed that proliferation-promoting effect of EHF were significantly attenuated upon HER2 depletion (Figure 9). Altogether, our data suggest that EHF promotes gastric tumorigenesis through transcriptionally regulating HER2 expression via binding to GGAA core sequence within its promoter.

## Discussion

In this study, we first provided strong evidences supporting the oncogenic activities of EHF in gastric cancer. First, *EHF* was frequently overexpressed and amplified in gastric cancers compared with matched non-cancerous gastric tissues. Second, *EHF* amplification (or overexpression) was significantly associated with poor clinical outcomes and may be used as a potential prognostic marker for gastric cancer patients. Third, knocking down EHF expression in gastric cancer cells significantly inhibited cell growth and invasiveness. Fourth, EHF was identified to be a new transcription factor of *HER2*, and also modulated the expression of *HER*3 and *HER4* in gastric cancer.

It has been well documented that genomic amplification is one of the major causes of oncogene overexpression in human cancers.^28, 35^ To identify the mechanisms that may contribute to *EHF* overexpression in gastric cancers, we examined *EHF* copy number and its mRNA/protein expression in gastric cancers, and demonstrated a significantly positive relationship between them, suggesting that *EHF* amplification is one of the major mechanisms of *EHF* overexpression in gastric cancers. In addition, our data showed that *EHF* amplification dramatically affected patient survival, implicating that it may be used as a potential prognostic marker for gastric cancer patients. However, our data also showed that *EHF* overexpression did not always coincide with its genomic amplification, suggesting that there are other possible mechanisms may contribute to its overexpression.

Although a previous study has reported frequent *EHF* amplification in gastric cancers,^36^ the role and mechanisms of EHF in gastric tumorigenesis remain totally unknown. We thus tested its oncogene function in gastric cancer by a series of *in vitro* and *in vivo* studies. As expected, EHF knockdown in gastric cancer cells showed significant growth-inhibitory effect by inhibition of cell proliferation and colony formation *in vitro* and tumorigenic potential in nude mice *in vivo*. EHF knockdown induced cell cycle arrest and apoptosis, and inhibited cell migration, invasion and EMT process in gastric cancer cells. We also demonstrated that knocking down EHF expression in gastric cancer cells significantly inhibited expression of *MMP-2*, *-7*, *-9* and *-14 g*enes, suggesting that the decrease in the metastasis-related phenotypes may be associated with suppression of expression or activities of MMPs. This is supported by previous studies that MMPs genes are key targets of ETS proteins and Ets-mediated induction of these genes contributes to the invasive and angiogenic phenotypes of malignant cells, such as PEA3, ETS1, and ETS2.^37, 38^ These findings further suggest that *EHF* may be a potential oncogene in gastric cancer cells.

As a member of the ETS family, downstream targets/pathways of EHF in gastric cancer remain to be identified. A previous study has reported that there is a conserved ETS-responsive element (GAGGAA) within a DNase I hypersensitive site in the proximal *HER2* promoter and demonstrated that it can be recognized by an ETS-immunoreactive factor in HER2-expressing breast cancer cells.^39^ However, although more than 10 different ETS factors have been found to be co-expressed with HER2 in cancer cells, only a few ETS family members such as Elf-1, PEA3 and ESE1 have been studied as potential *HER2* transactivators.^34, 40, 41, 42^ The core GGAA/T motif (ETS binding site, EBS) was found in the *HER2* promoter by using MatInspector online software. To be consistent with this, we found that there were positive correlations between the expression of *EHF* and HER receptors in a cohort of gastric cancers including *EGFR*, *HER2* and *HER3*, particularly *HER2*, in a cohort of gastric cancers, as supported by the information from TGCA database. Down-regulating EHF expression in gastric cancer cells significantly reduced mRNA expression of *HER2-4*. However, the luciferase reporter gene assays demonstrated that ectopic expression of EHF only enhanced promoter activity of *HER2*, but not *HER3* and *HER4*, in gastric cancer cells. These data suggest that *HER2* may be a potential downstream target of EHF, whereas *HER2* and *HER4* may be indirect targets of EHF, as supported by the ChIP assay. Accordingly, EHF knockdown remarkably inhibited the activity of their downstream signaling pathways such as the MAPK/Erk and PI3K/Akt pathways. Collectively, EHF may be a new transcription factor for *HER2* in gastric cancer by binding to a functional EBS within its promoter, and promotes gastric tumorigenesis by activating HER family of receptor tyrosine kinase.

EHF has been demonstrated to be a potential tumor-suppressor in prostate cancer and ESCC,^19, 22, 23^ whereas our data suggest that *EHF* may be a functional oncogene in gastric cancer. We thus speculate that EHF derived from different types of cells or tissues has strikingly distinct functional activities in tumorigenesis. It is the fact that the DNA binding domains of ETS factors are very similar, thus their specific roles in tumorigenesis are largely dependent on other factors including interaction with other nuclear factors such as transcription factors, co-activators or co-repressor.^43, 44^ Moreover, the activities of ETS proteins are also regulated by post-translational modifications such as phosphorylation, acetylation, sumoylation, ubiquitinylation and glycosylation.^45^

In summary, we found frequent overexpression and amplification of *EHF* in gastric cancers and revealed a strong association of *EHF* overexpression/amplification with poor patient outcomes. Moreover, our data support an oncogenic role of EHF as a *HER2* transcription factor and the modulator of *HER3* and *HER4* in gastric tumorigenesis. A better understanding of the physiologic and pathologic function of EHF will significantly improve our knowledge of the pathogenesis of gastric cancer, and targeting this frequently overexpressed/amplified oncogene may elucidate the effective treatment of this cancer in the future.

## Materials and Methods

### Clinical samples

A cohort of primary cancer tissues and matched non-cancerous tissues or benign tumors were obtained from patients who underwent surgery at the First Affiliated Hospital of Xi'an Jiaotong University, including gliomas, gastric, lung and thyroid cancers. Moreover, a total of 131 paraffin-embedded gastric cancer tissues were randomly obtained from the First Affiliated Hospital of Xi'an Jiaotong University between January 1999 and December 2005, with a follow-up period of 10 years after the surgery. Normal controls from 37 patients with chronic gastritis who underwent endoscopic biopsy were also obtained from the same hospital. Informed consent was obtained from each patient before the surgery. All patients did not receive chemotherapy and radiotherapy before the surgery, and all sections were histologically examined by a senior pathologist at Department of Pathology of the Hospital based on World Health Organization criteria. Clinicopathological data were collected from the patients' files or by interview with the patients or their relatives and were summarized in Supplementary Table 1. The study protocol was approved by the Human Ethics Committee of the First Affiliated Hospital of Xi'an Jiaotong University.

### Cell culture and short interfering RNA (siRNA) cell transfection

Human gastric cancer cell lines AGS, BGC823, MGC803 and SGC7901, human immortalized gastric mucosal epithelial cell line GES-1 and embryonic kidney cell line 293T were used in this study. These cells were maintained in RPMI medium 1640 or Dulbecco's modified Eagle medium (Gibco, Grand Island, NY) with 10% fetal bovine serum (FBS; Hyclone, Logan, UT). Oligonucleotides of target-specific and control siRNAs were obtained from GenePharma (Shanghai, P.R. China) and the sequences were presented in Supplementary Table 3. Cells were transfected at 70% confluence using Lipofectamine 3000 (Invitrogen, Grand Island, NY), with a final siRNA concentration of 50 nM. Specific oligonucleotides with maximal knockdown efficiency were selected among three different sequences until use. All silencing experiments were performed in three replicates.

### EHF expression plasmid construction

To construct EHF expression plasmid, total RNA from GES-1 cell line was isolated by TRIzol Reagent following manufacturer's instruction (Invitrogen, Grand Island, NY). The cDNA was reverse transcribed by using PrimeScript™ II 1st Strand cDNA Synthesis Kit (Takara Inc., Dalian, P.R. China). The full-length open reading frame (ORF) of human ESE with or without stop codon TGA was amplified and then cloned into pcDNA3.1(−)A mammalian expression vector with a Myc-His tag (Invitrogen, Grand Island, NY), which was designated as pcDNA3.1/myc-His(−)A-EHF or pcDNA3.1(−)A-EHF.

### RNA extraction and quantitative RT-PCR (qRT-PCR)

Total RNA from tissues and cell lines were extracted using Trizol reagent (Takara Inc., Dalian, P.R. China) following the manufacturer's protocol. The cDNA was synthesized with 500 ng total RNA by using PrimeScript RT reagent Kit (Takara Inc., Dalian, P.R. China). Quantitative RT-PCR (qRT-PCR) was carried out on a CFX96 Thermal Cycler DiceTM real-time PCR system (Bio-Rad Laboratories, Inc., CA) using SYBR Premix Ex TaqTM (Takara Inc., Dalian, P.R. China). The mRNA expression of the indicated genes was normalized to 18S rRNA cDNA. Each sample was run in triplicate. The primer sequences were presented in Supplementary Table 4.

### Tissues and DNA preparation

Paraffin-embedded serial sections were cut at intervals of 5 *μ*m. One of sections was stained by hematoxylin and eosin (H&E) and was marked as a tumor representative tissue by a senior pathologist for gastric cancer. Tumor tissues were isolated by manual microdissection under an inverted microscope using the marked H&E section as target tissue identification. DNA was extracted from isolated tissues as previously described.^28^ The fresh gastric tissues were collected and immediately frozen in liquid nitrogen and stored at −80 °C prior to DNA extraction. DNA was then isolated using standard phenol/chloroform protocol.

### Copy number analysis

Real-time quantitative PCR was performed to analyze *EHF* copy number in gastric cancers and control subjects as described previously.^28, 46^ The TaqMan probe for *EHF* was 5′-6FAM-AAC CTG CCT TTCTGC TTT TCA TCA GAC CC-TAMRA-3′, and the primers were 5′-CCT ATC TTTGCT GTG ACT TAG ATC ATT AG-3′ (forward) and 5′-CGG ATG AAT TCCCAT AAG TGA GT-3′ (reverse). The TaqMan probe and primers for *β-actin* were described previously.^28^ Each sample was run in triplicate, and *β-actin* was run in parallel to normalize the input DNA. Standard curves were established using serial dilutions of normal leukocyte DNA. *EHF* amplification was defined by a copy number ≥4.

### Fluorescence *in situ* hybridization

The FISH analysis was performed on formalin-fixed, paraffin-embedded gastric cancer tissues and matched non-cancerous tissues using the EHF DNA probe/CEN11 probe mixture (Exon Biotechnology Inc, Guangzhou, P.R. China). Briefly, the paraffin-embedded tissue slides were deparaffinized through xylene, and rehydrated in an ethanol series (100, 85 and 70%), and treated with protenase K solution (200 *μ*g/ml) and pepsin (0.005% in 0.01 M HCl solution) at 37 °C, respectively. The slides were then dehydrated in an ethanol series (70, 85 and 100%), and the probe mixture was added to the slides and immediately covered by coverslips and sealed the edges with rubber cement. The slides were subsequently denatured at 85 °C for 5 minutes and incubated at 37 °C overnight. After hybridization, the slides were washed in 2 × SSC, 2 × SSC/0.1% NP-40 buffer at 37 °C for 5 min each, and were counterstained with DAPI antifade solution. FISH signals in 20–30 cells for each specimen were counted, and the criteria for gene amplification were defined when FISH signals were detected by tested probes compared with control probes ≥1.5. Fluorescence images were captured with Olympus IX71 microscope (Olympus, Tokyo, Japan), which enables simultaneous detection of both FITC and Texas Red fluorescence. The color mergence was performed using ImageJ image software (ImageJ version 1.44p, NIH, MD).

### Immunohistochemistry

Immunohistochemical analysis was performed as previously described.^28, 46^ Briefly, after dewaxing in xylene and rehydrating in a gradient concentration of ethanol, the paraffin-embedded tissue slides were incubated in 0.3% hydrogen peroxide in distilled water to block endogenous peroxidase activity, and treated with an antigen retrieval method by heating, and were then incubated with rabbit anti-EHF antibody (Abcam, Inc; 1 : 100) or mouse anti-Ki67 antibody (BD Biosciences, Inc; 1 : 200) overnight at 4 °C. Subsequently, the slides were incubated with biotinylated goat anti-rabbit IgG or goat anti-mouse IgG (ZSGB-bio, Beijing, P.R. China). Immunodetection was performed with the Streptavidin-Peroxidase system (ZSGB-bio, Beijing, P.R. China) according the manufacture's protocol. After washing, diaminobenzidine and hematoxylin were respectively added to detect immunoreactive proteins. EHF protein expression was scored in double-blinding way (ie, without knowing the EHF copy number of the case), and 0, 1, 2, 3 reprents negative, weak positive, positive and strong positive, respectively.

### Western blot analysis

Cells were lysed in prechilled RIPA buffer containing protease inhibitors. The protein lysates were separated on SDS–PAGE and then transferred to PVDF membranes (Roche Diagnostics, Mannheim, Germany). The membranes were blocked for 2 h in 5% bovine serum albumin (BSA) in 1 × TBS-T (0.5% Tween-20) and incubated with the indicated primary antibodies, including anti-EHF (Abcam, Inc), anti-total-Erk1/2 (Abcam, Inc), anti-phospho-Erk1/2 (Epitomics, Inc), anti-phospho-AktSer473 (Bioworld Technology, co, Ltd), anti-total-Akt (Bioworld Technology, co, Ltd), anti-HER2 (Sino Biological, Inc), anti-HER3 (Sino Biological, Inc), anti-HER4 (Sino Biological, Inc), anti-E-cadherin (Epitomics, Inc), anti-Vimentin (Epitomics, Inc) and anti-GAPDH (Abgent, Inc). The membranes were then incubated with species-specific HRP-conjugated secondary antibodies from ZSGB-BIO, and immunoblotting signals were visualized using the Western Bright ECL detection system (Advansta, CA).

### Cell proliferation, cell cycle and apoptosis assays

Cell proliferation, colony formation, cell cycle and apoptosis assays were similarly performed as previously described.^46^

### *In vivo* tumorigenicity

Four- to five-week-old male athymic nude mice were purchased from SLAC laboratory Animal Co., Ltd. (Shanghai, PR. China) and housed in a specific pathogen-free (SPF) environment. The mice were randomly divided into two groups (six mice per group). Tumor xenografts were established by subcutaneously injecting 4 × 10^6^ BGC823 cells transfected with the indicated siRNAs into the right armpit region of nude mice. From day 3 post-injection, tumor size was measured every 2 days. Tumor volumes were calculated by the formula (length × width^2^ × 0.5). The mice were sacrificed after 15 days. Tumors were harvested and weighted. Tumors obtained from representative animals were embedded in paraffin, sectioned at 4 *μ*m, and stained with H&E. Ki-67 staining was used to evaluate cell proliferation. All experimental procedures were approved by the Animal Ethics Committee of Xi'an Jiaotong University.

### Cell migration and invasion assays

Cell migration and invasion assays were similarly performed as previously described.^46^

### Immunofluorescence staining

The process of IF staining was similarly performed as described previously.^46^

### Dual-luciferase reporter assay

To construct luciferase reporter plasmids, the promoter regions of *HER2-4* genes were amplified from genomic DNA of BGC823 cells. The amplification products were digested with restriction enzymes and inserted into pre-digested pGL3-Basic luciferase vector (Promega Corp., Madison, WI, USA) to produce the luciferase reporter plasmids pGL3-HER2-Luc, pGL3-HER3-Luc and pGL3-HER4-Luc. All of the constructs were verified by Sanger sequencing. The primers for plasmid constructs were presented in Supplementary Table 5.

To test the promoter activity of *HER2-4* genes modulated by EHF, BGC823 or 293T cells were transfected with pcDNA3.1(-)A-EHF or empty vector in six-well plates and were cotransfected with pGL3-HER2-Luc, pGL3-HER3-Luc or pGL3-HER4-Luc, and pRL-TK plasmids (Promega Corp., Madison, WI, USA) using Lipofectamine 3000 (Invitrogen, Grand Island, NY, USA). The pRL-TK plasmid, containing Renilla luciferase, was used to normalize transfection efficiency. Cells were collected 36 h post-transfection, and luciferase activities were analyzed on EnSpire Multimode Plate Reader (PerkinElmer, Waltham, MA, USA) using the dual-luciferase reporter assay system (Promega Corp., Madison, WI, USA) according to the manufacturer's instructions. Data were expressed as relative luciferase activity (Firefly luciferase activity/Renilla luciferase activity). Each experiment was performed in triplicate.

### Chromatin immunoprecipitation assay

The ChIP assay was used to evaluate transcription factor EHF binding to its target DNA by using the Pierce Magnetic ChIP Kit (Pierce Biotechnology, Rockford, IL, USA). In brief, BGC823 cells were transfected with pcDNA3.1/myc-His(-)A-EHF and empty vector. After 2 days, the indicated cells (1 × 10^7^cells) were cross-linked with formaldehyde (final concentration 1% vol/vol) and the cross-linking reaction was stopped by the addition of glycine. The harvested cells were then lysed and digested by using membrane extraction buffer and MNase digestion buffer, and the chromatin was sonicated by using sonics VCX-130PB (Sonics & Materials, Inc., Newtown, CT, USA). Next, 10% of the chromatin from each lysate was saved as an input control. The remaining chromatin was immunoprecipitated by using mouse monoclonal anti-Myc tag, clone 4A6 antibody (Millipore, Temecula, CA, USA). The same amount of non-specific IgG was used as control. Immunoprecipitated protein DNA complex was then captured with ChIP Grade Protein A/G Magnetic Beads. After reversal of the cross-link, digestion of proteins with proteinase K and DNA recovery, the DNA fragments were used as templates for qRT-PCR analysis using the primers presented in Supplementary Table 6, and the data were normalized by respective 5% input. Each experiment was performed in triplicate.

### Electrophoretic mobility shift assays

EHF protein was obtained when 1 *μ*g of pcDNA3.1(-)A-EHF or empty vector as DNA template was used according to the protocol of the TNT T7/SP6 Coupled Reticulocyte Lysate System (Promega Corp., Madison, WI, USA). EMSA was performed by using LightShift Chemiluminescent EMSA Kit (Pierce Biotechnology, Rockford, IL, USA) following the manufacturer's protocol. The binging reaction mixtures contained 2 *μ*l of the translation products, 100 fmol of biotin-labeled oligonucleotide probes (Sangon, Shanghai, China), 2.5% glycerol, 5 mM MgCl2, 50 ng/*μ*l Poly (dI-dC) and 1% NP-40 were incubated in binding buffer at room temperature for 20 min. Unlabeled wild type or mutant oligonucleotides (10 pmol) were incubated with the translation products at room temperature for 15 min prior to the addition of biotin-labeled probes. The mixtures containing loading buffer were separated on a 6% non-denaturing polyacrylamide gel in 0.5 × TBE buffer at 100 V, and oligonucleotides were electrophoretic transferred to a nylon membrane (Roche Diagnostics, Mannheim, Germany). After cross-linking with HL-2000 HybriLinker Hybridization Oven (UVP, Upland, CA, USA), the membrane was detected using a LightShift Chemiluminescent EMSA Kit. The sequences of the double-stranded oligonucleotides used to detect the DNA-binding activity of EHF were presented in Supplementary Table 7.

### Statistical analysis

Statistical analysis was performed using the SPSS statistical package (11.5, Chicago, IL, USA). *EHF* expression and amplification in cancer tissues and control subjects were compared by the paired-samples *t*-test, Mann–Whitney *U* test or Wilcoxon Signal-Rank Test. The correlation between *EHF* amplification and clinicopathological characteristics of gastric cancer patients was analyzed by Fisher's exact test or Pearson's *χ*^2^-test. Multivariate models that adjusted for the most important covariates were analyzed by logistic regression test. Survival curves were constructed according to the Kaplan–Meier method and statistical analysis was performed using the log-rank test. Univariate survival analysis was performed to investigate the effect of *EHF* amplification on the survival of gastric cancer patients. Multivariate cox regression analysis was used to examine the effect of *EHF* amplification on survival of independently of gender, age, differentiation and tumor stage. *P*<0.05 were considered significant.

## Figures and Tables

**Table 1:** Prognostic value of *EHF* amplification in univariate and multivariate Cox regression analysis (*n*=131)

**Characteristics**	**Univariate analysis**		**Multivariate analysis**	
	**HR** **(95% CI)**	P***-value***	**HR** **(95% CI)**	P-***value***
*EHF* amplification	2.593 (1.583–4.249)	0.0002	2.426 (1.474–3.991)	<0.0001
Male *versus* female	0.886 (0.483–1.626)	0.696	—	—
Age^a^	1.334 (1.052–1.692)	0.017	1.210 (0.949–1.543)	0.125
Differentiation^b^	1.439 (0.874–2.369)	0.152	—	—
TNM stage^c^	2.814 (1.940–4.081)	<0.0001	2.748 (1.872–4.043)	<0.0001

Abbreviations: CI, confidence interval; HR, hazard ratio

a Age (per 10 years)

b Differentiation (well or moderate; poor or no differentiation)

c TNM stage (I; II; III; IV)

## Abbreviations

ChIP: chromatin immunoprecipitation
qRT-PCR: quantitative RT-PCR
EBS: ETS-binding site
EMSA: electrophoretic mobility shift assay
EMT: epithelial–mesenchymal transition
ESCC: esophageal squamous cell carcinoma
ETS: E26 transformation-specific
FISH: fluorescence *in situ* hybridization
H&E: hematoxylin and eosin
HER: human epidermal growth factor receptor
IF: immunofluorescence
IHC: immunohistochemistry
MMPs: matrix metalloproteinases
PI3K: phosphatidylinositol-3-kinase
