# Supplementary figures and images for: Increased expression of EHF via gene amplification contributes to the activation of HER family signaling and associates with poor survival in gastric cancer
Source: Cell Death Dis. 2016 Oct 27;7(10):e2442–. doi: 10.1038/cddis.2016.346 (PMC5134001; doi:10.1038/cddis.2016.346)

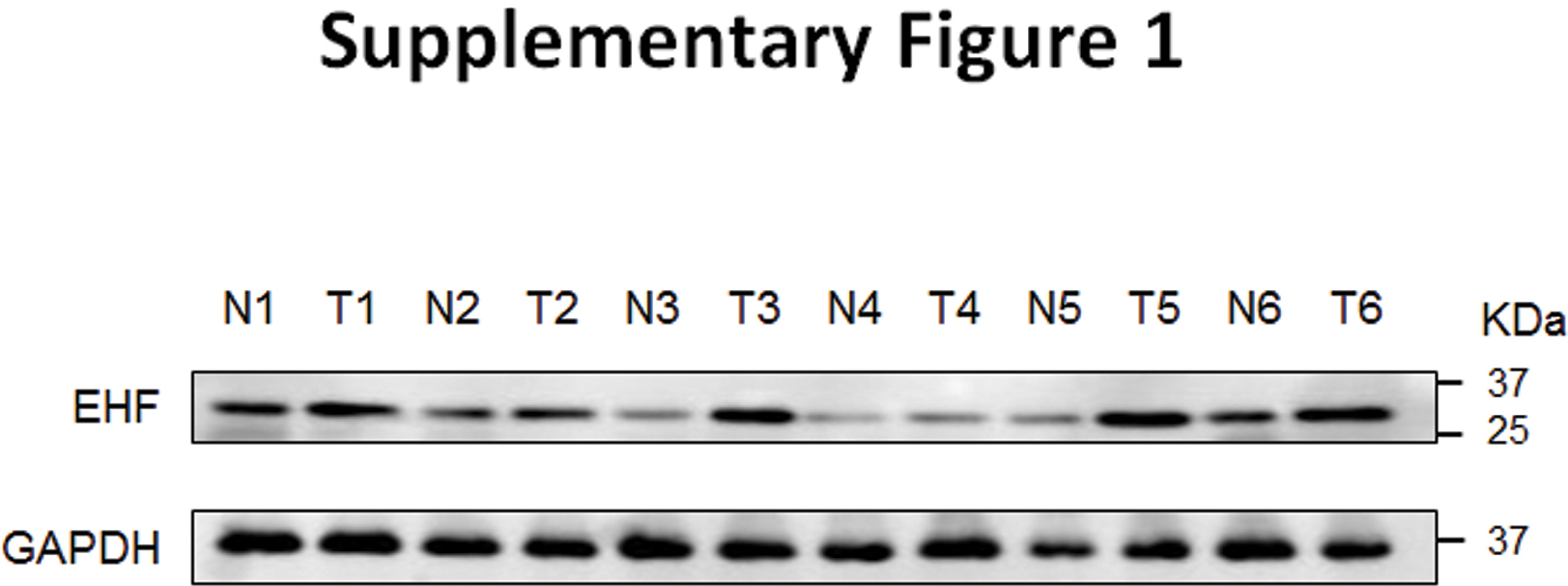

Supplement: Supplementary Figure 1 [file cddis2016346x1.tif]

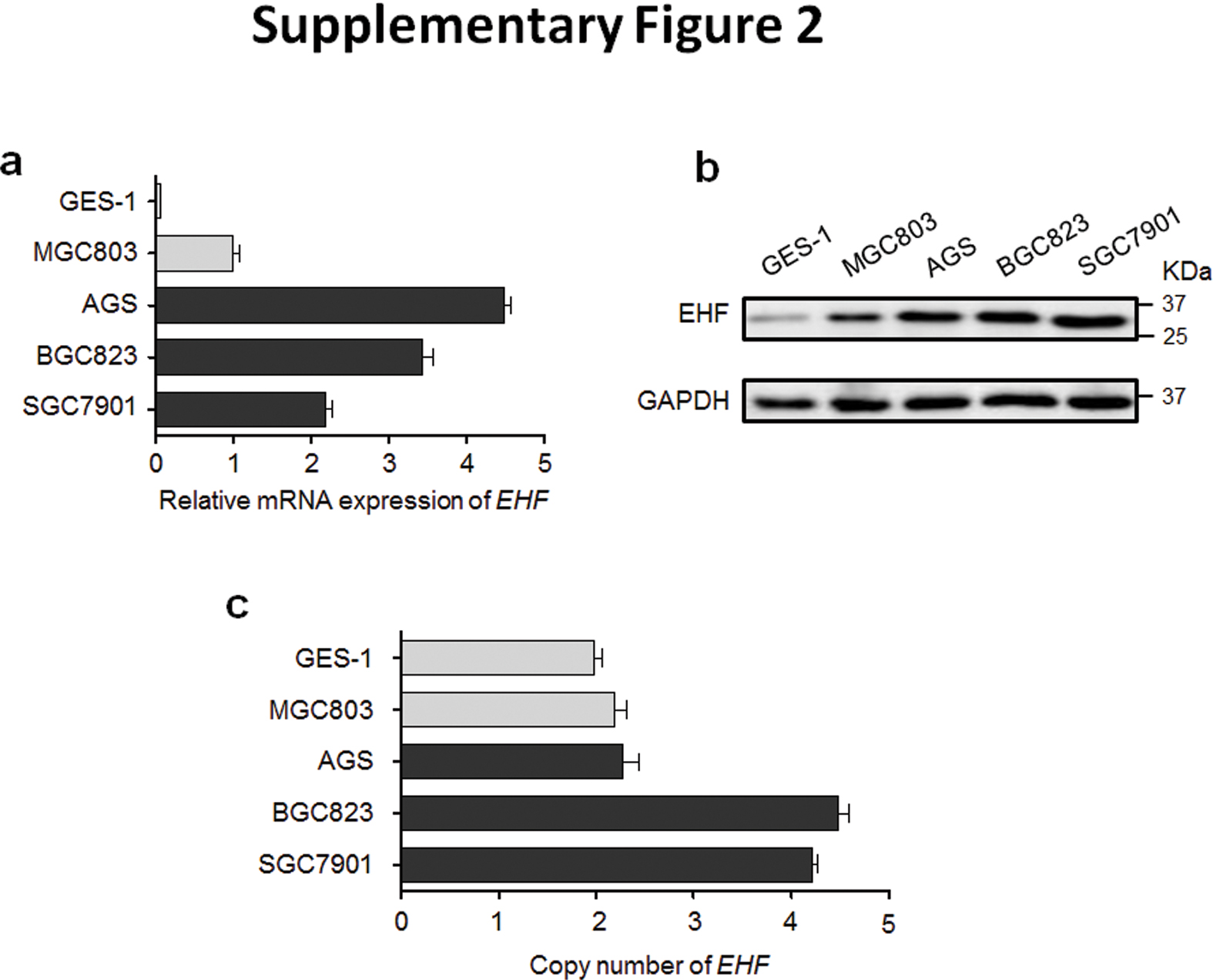

Supplement: Supplementary Figure 2 [file cddis2016346x2.tif]

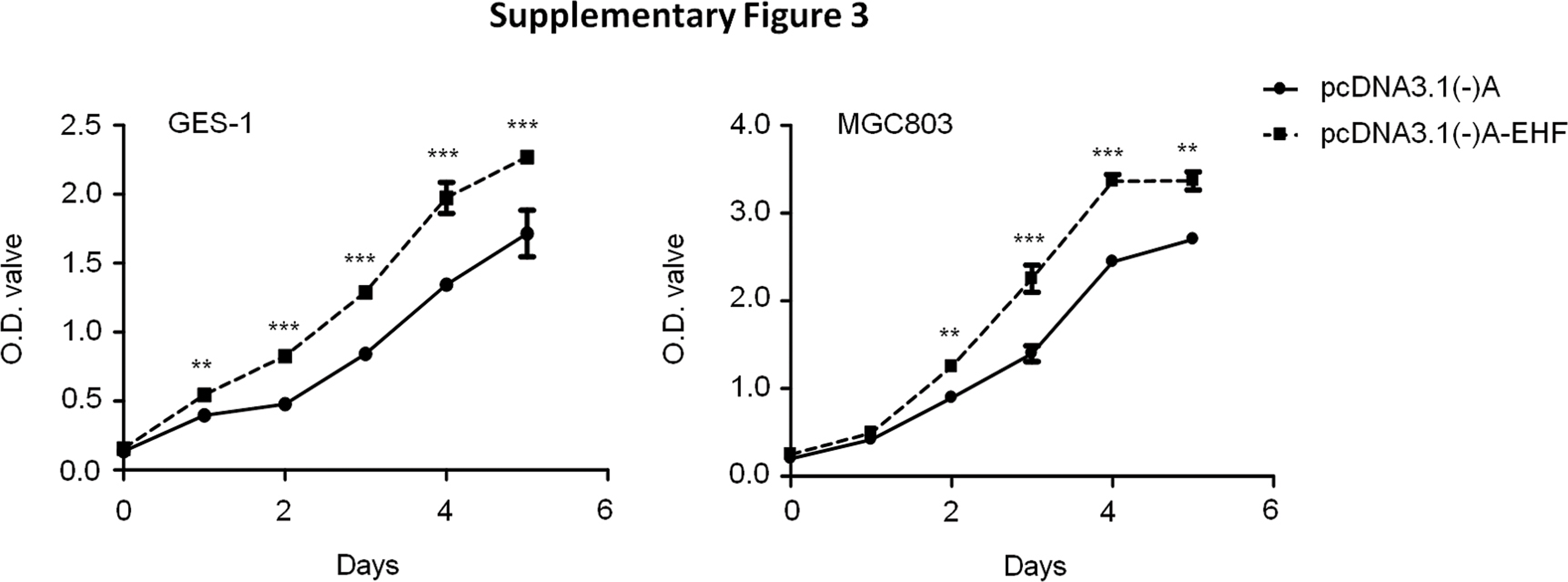

Supplement: Supplementary Figure 3 [file cddis2016346x3.tif]

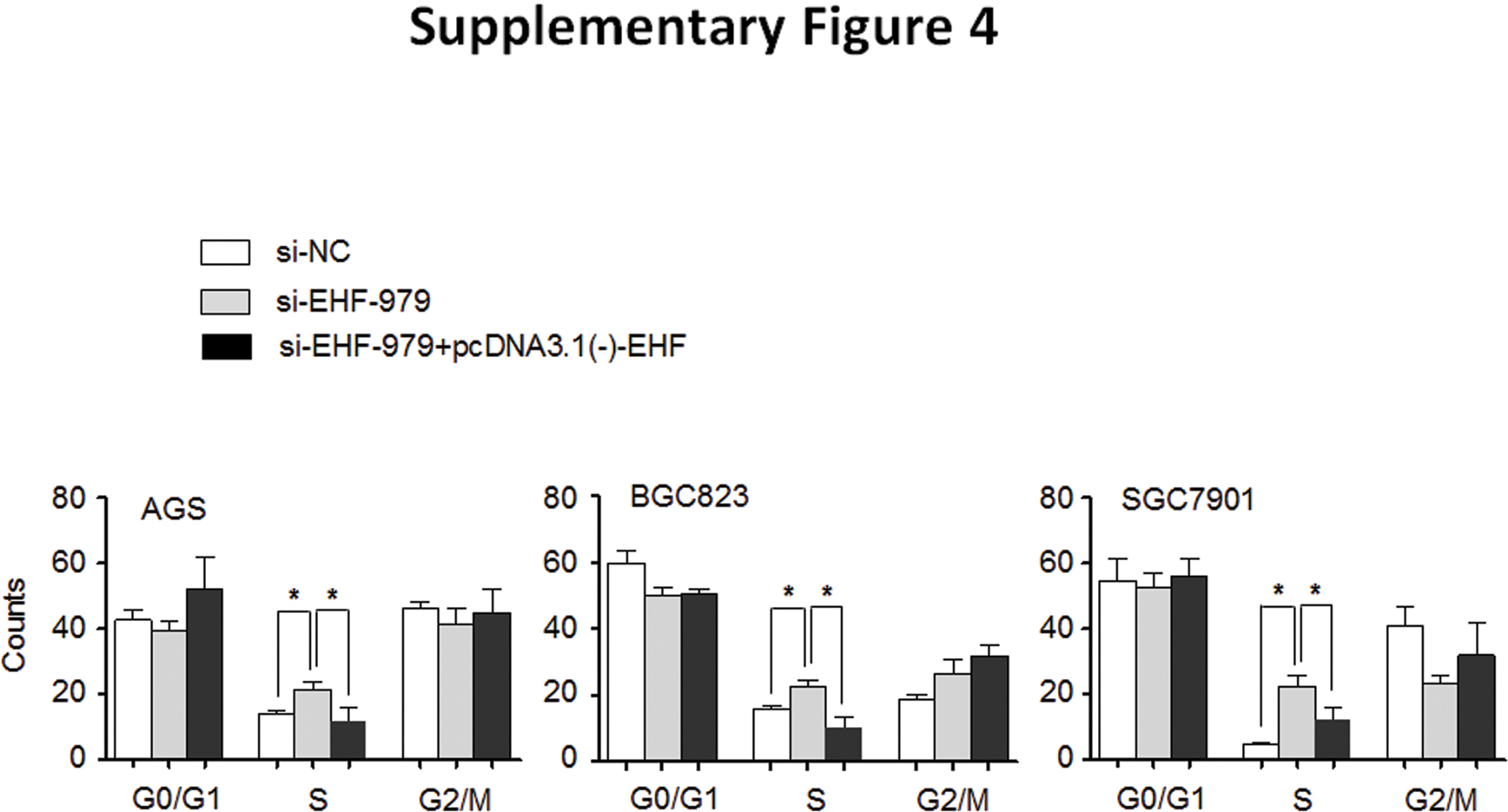

Supplement: Supplementary Figure 4 [file cddis2016346x4.tif]

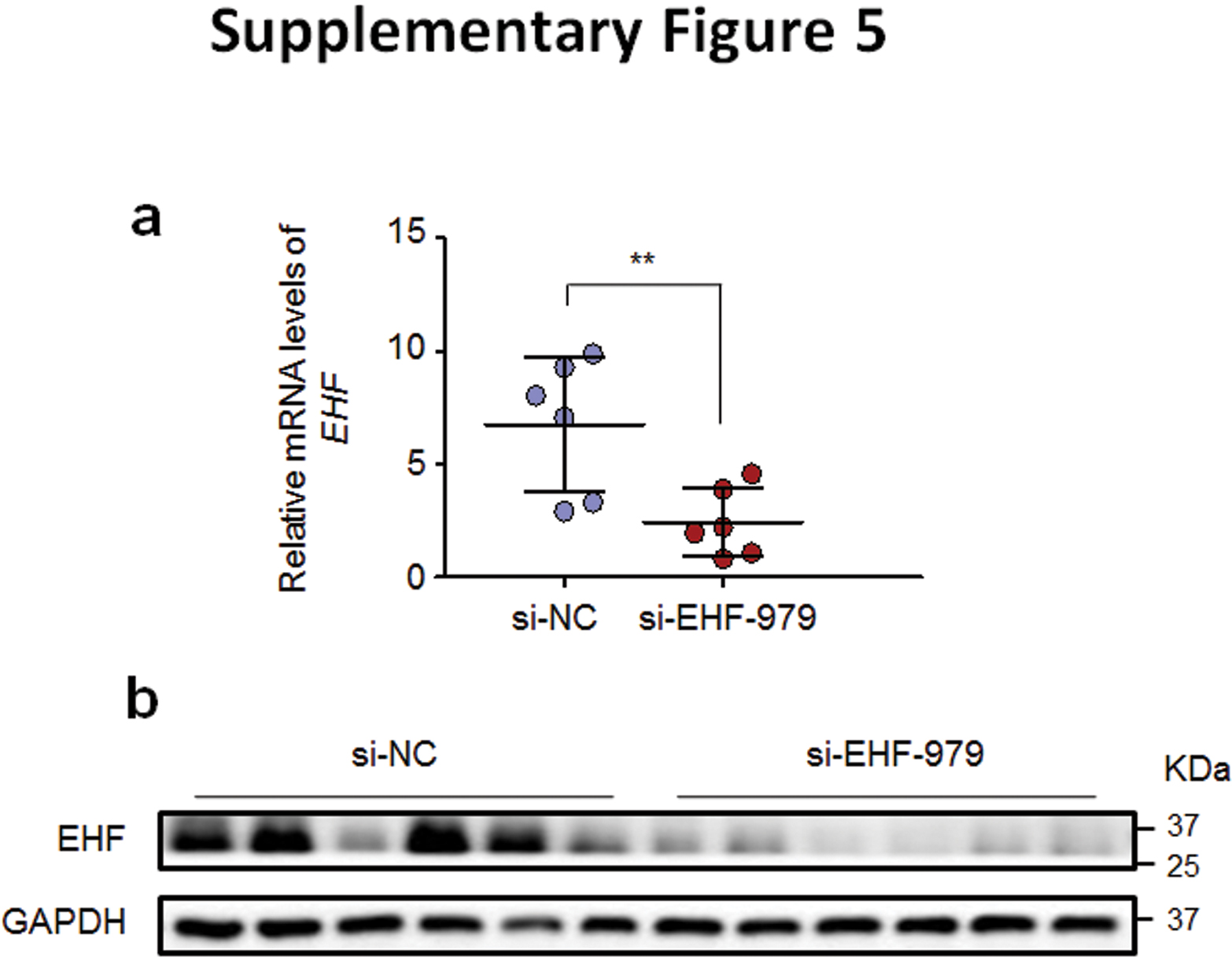

Supplement: Supplementary Figure 5 [file cddis2016346x5.tif]

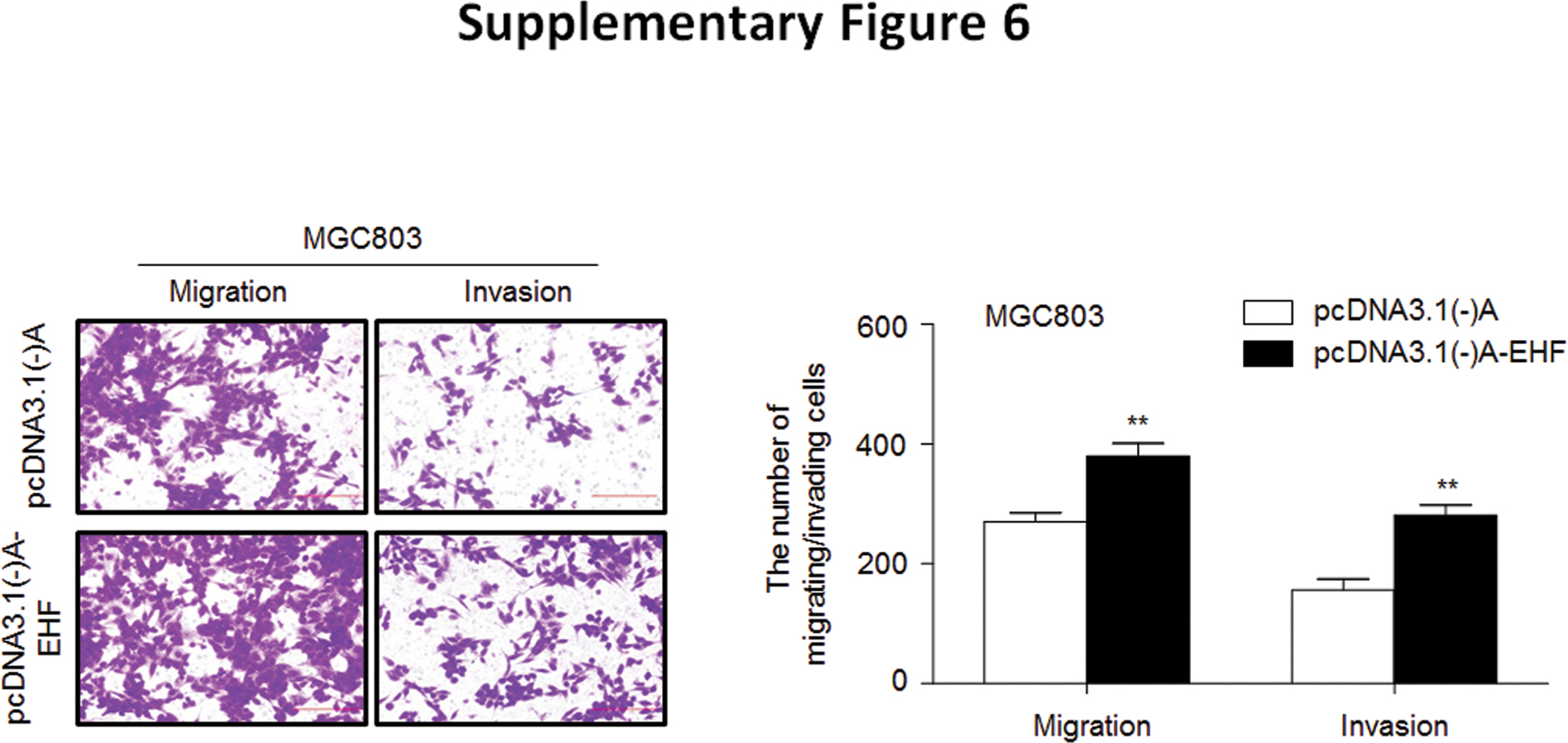

Supplement: Supplementary Figure 6 [file cddis2016346x6.tif]

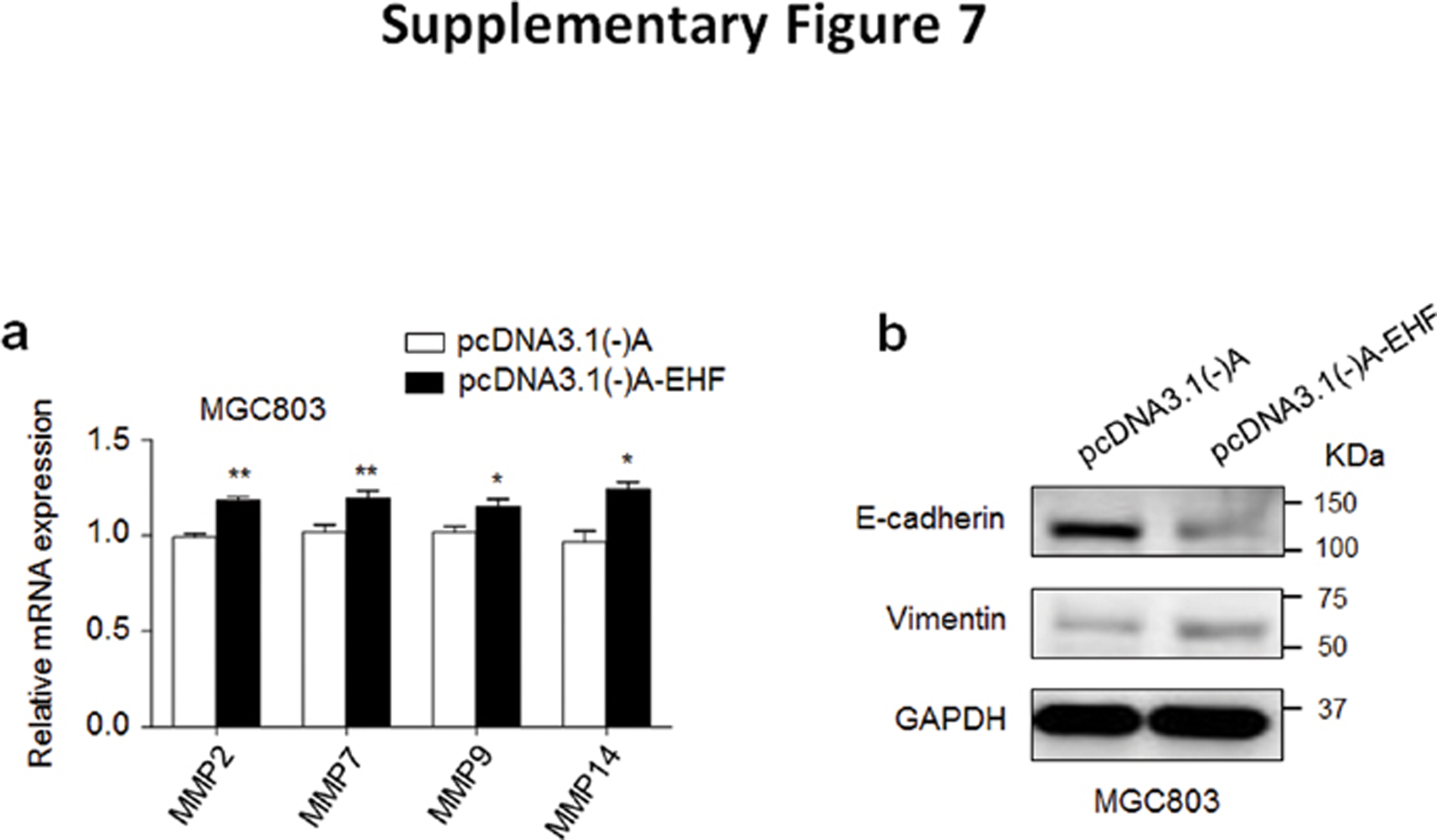

Supplement: Supplementary Figure 7 [file cddis2016346x7.tif]

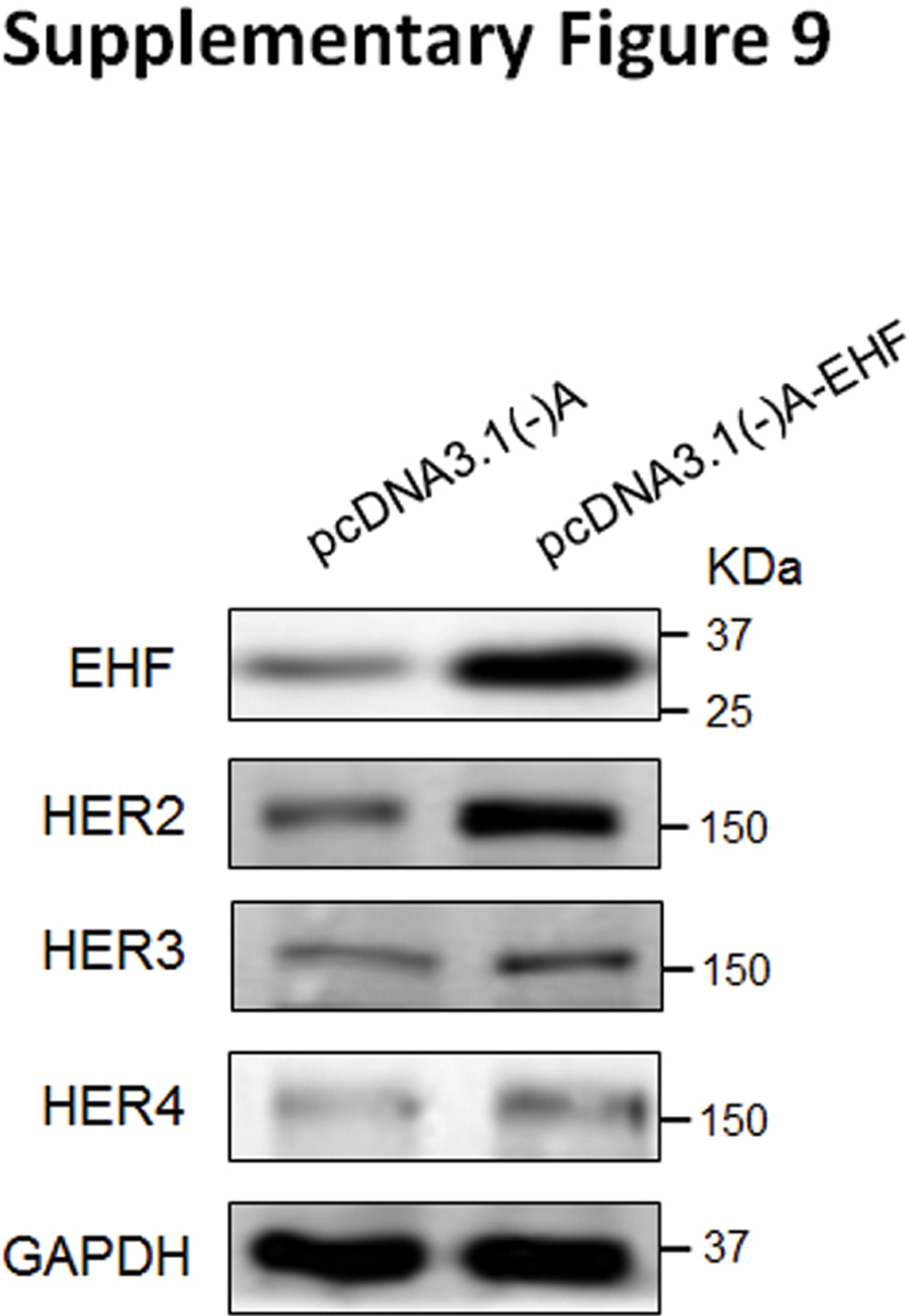

Supplement: Supplementary Figure 9 [file cddis2016346x9.tif]

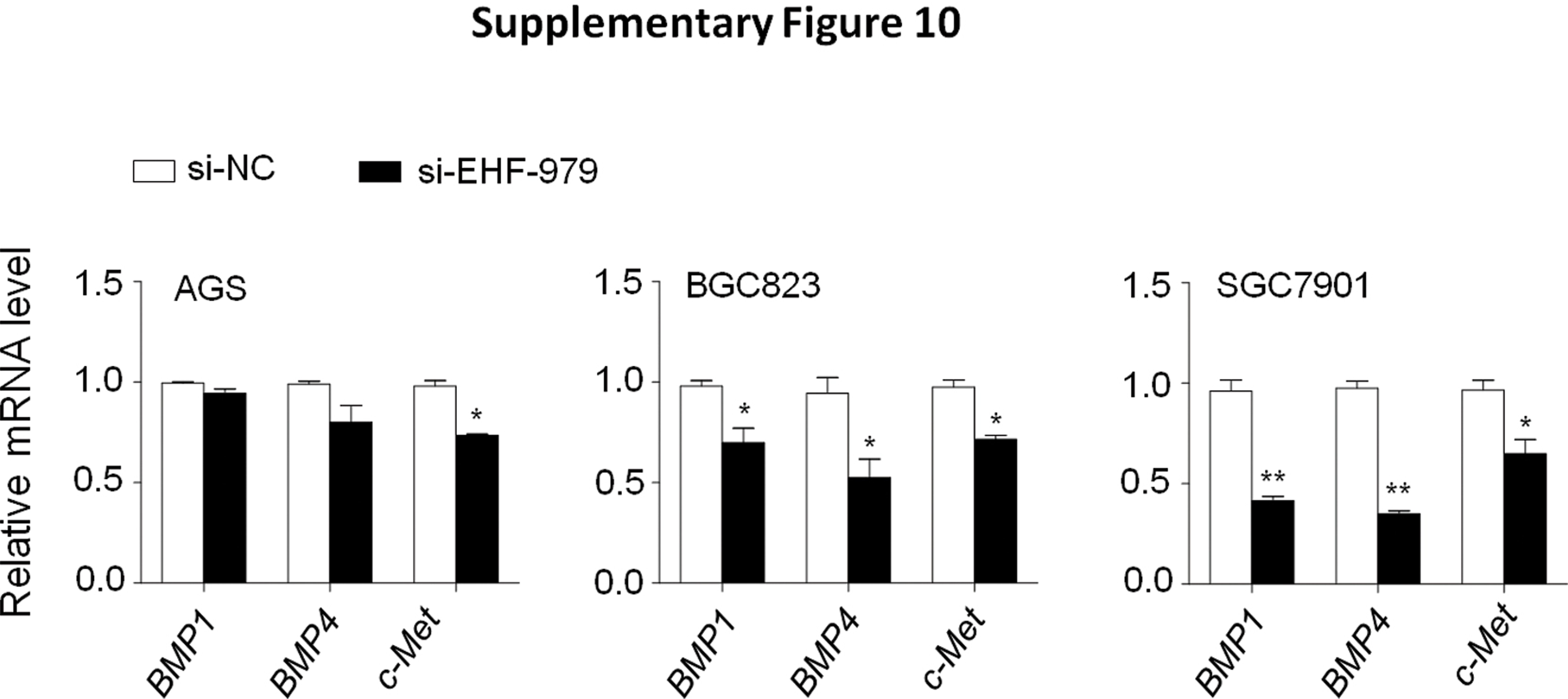

Supplement: Supplementary Figure 10 [file cddis2016346x10.tif]

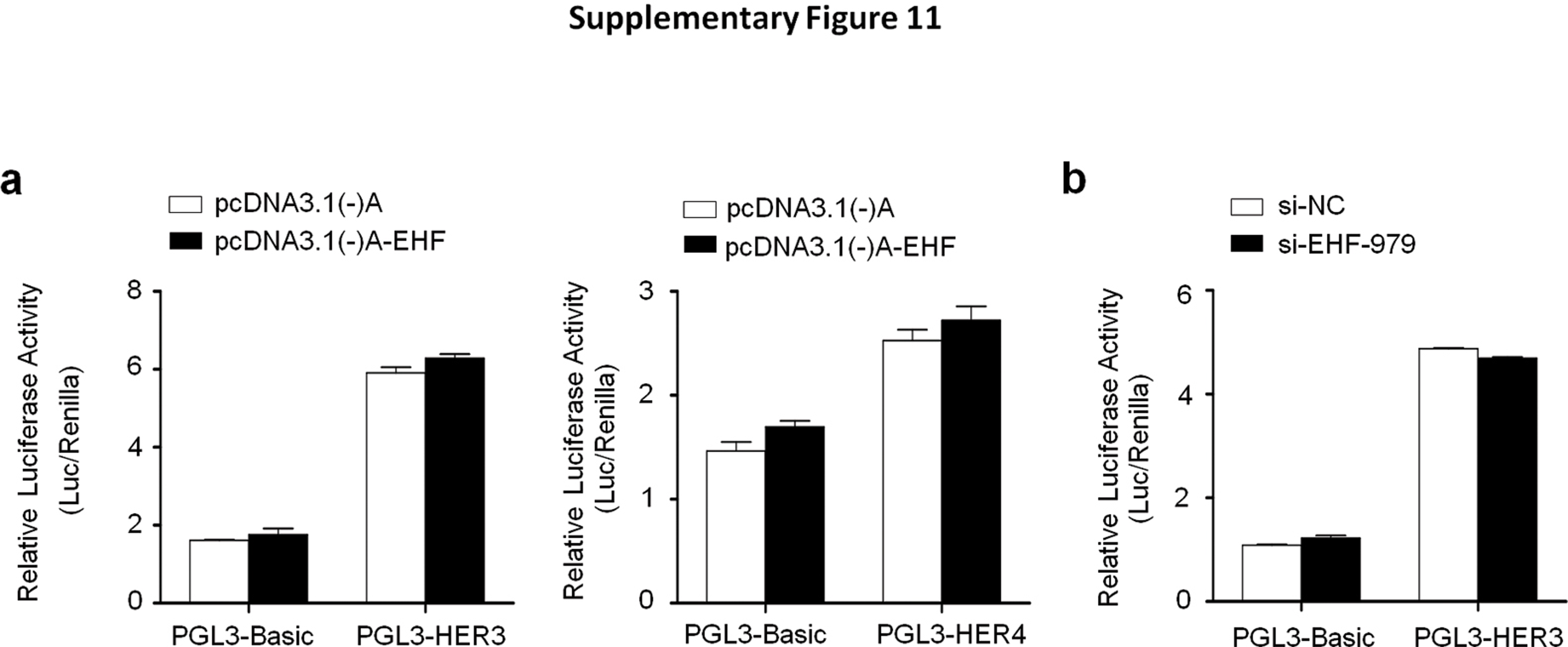

Supplement: Supplementary Figure 11 [file cddis2016346x11.tif]
