# Supplementary material for: Increased expression of EHF via gene amplification contributes to the activation of HER family signaling and associates with poor survival in gastric cancer
Source: Cell Death Dis. 2016 Oct 27;7(10):e2442–. doi: 10.1038/cddis.2016.346 (PMC5134001; doi:10.1038/cddis.2016.346)
Supplement: Supplementary Figures [file cddis2016346x12.doc]

**Supplementary Figure Legends**

**Supplementary Figure 1.** Western blot analysis was performed to evaluate EHF expression in six pairs of gastric cancers and normal gastric tissues. GAPDH was used as loading control.

**Supplementary Figure 2.** (**a**, **b**) mRNAa nd protein levels of EHF were determined in GES-1, AGS, BGC823 and SGC7901 cells by qRT-PCR and western blot assays. (**c**) Copy number of *EHF* was analyzed in the indicated cells by qPCR approach. *18S* rRNA was used as a normalized control for qRT-PCR. GAPDH was used as loading control in western blot analysis. *β-actin* was used to normalize the input DNA in copy number analysis.

**Supplementary Figure 3.** Ectopic expression of EHF in GES-1 and MGC803 cells promoted cell proliferation as compared with empty vector. Data were presented as mean ± SE. **, *P* <0.01, ***, *P* <0.001.

**Supplementary Figure 4.** Effect of EHF depletion on cell cycle is reversed by exogenous overexpressing EHF in AGS, BGC823 and SGC7901 cells. *, *P* <0.05.

**Supplementary Figure 5.** qRT-PCR (**a**) and western blot (**b**) assays were performed to test EHF expression in xenograft tumours. *18S* rRNA was used as a normalized control for qRT-PCR. GAPDH was used as loading control for western blot. **, *P* <0.01,

**Supplementary Figure 6.** Overexpressing EHF in GES-1 and MGC803 cells promotes cell migration and invasion. The representative images of migrated/invaded cells (left panels). Histograms represent means ± SE of cell numbers from three independent assays (right panels). Magnification for each set: ×200. Scale bars, 50 µm.

**Supplementary Figure 7.** (**a**) qRT-PCR assay was performed to evaluate the effect of EHF overexpression on the expression of metastasis-related genes *MMP-2*, *-7*, *-9* and *-14* in MGC803 cells. Expression levels of these genes were normalized with *18S* rRNA levels. (**b**) Western blot was used to evaluate the effect of EHF overexpression on the expression of E-cadherin and Vimentin in MGC803 cells. GAPDH was used as loading control. Data were presented as mean ± SE. *, *P*<0.05; **, *P* <0.01.

**Supplementary Figure 8.** Association of *EHF* expression with the expression of *EGFR*, *HER2*, *HER3* and *HER4* in TCGA (n =384) data set.

**Supplementary Figure 9.** Western blot assay was performed to test the effect of EHF overexpression on HER2-4 in MGC803 cells. GAPDH was used as loading control.

**Supplementary Figure 10.** qRT-PCR assay was performed to test the effect of EHF knockdown on the expression of *BMP1*, *BMP4* and *c-Met* in gastric cancer cells. Expression levels of these genes were normalized with *18S* rRNA levels. Data were presented as mean ± SE. *, *P* <0.05; **, *P* <0.01.

**Supplementary Figure 11.** The luciferase reporter gene assay was performed to evaluate the effect of ectopic expression of EHF on promoter activity of *HER3* and *HER4* (**a**) and the effect of EHF knockdown on promoter activity of *HER3* (**b**) in BGC823 cells. The ratio of the Luc/Renilla activity is shown as means ± SE of three independent assays. **, *P* <0.01.
