# Supplementary material for: Increased expression of EHF via gene amplification contributes to the activation of HER family signaling and associates with poor survival in gastric cancer
Source: Cell Death Dis. 2016 Oct 27;7(10):e2442–. doi: 10.1038/cddis.2016.346 (PMC5134001; doi:10.1038/cddis.2016.346)
Supplement: Supplementary Tables [file cddis2016346x13.doc]

**Supplementary Tables**

**Supplementary Table 1**. Association of *EHF* amplification with clinicopathologic characteristics in gastric cancer

| **Variables** | ***EHF* amplification (n =131)** | | |
| --- | --- | --- | --- |
|  | **Yes** | **No** | ***P*** |
| No. of patients | 55 | 76 |  |
| Gender |  |  |  |
| Male | 42 | 60 | 0.725 |
| Female | 13 | 16 |  |
| Age, years |  |  |  |
| Mean±SD | 59.18±13.96 | 59.63±12.49 |  |
| ≤50 | 14 | 19 | 0.476 |
| 50-60 | 10 | 19 |  |
| 60-70 | 19 | 27 |  |
| >70 | 12 | 11 |  |
| Tumor localization |  |  |  |
| Gastric cardia | 13 | 22 | 0.386 |
| Gastric body | 14 | 21 |  |
| Gastric antrum | 28 | 33 |  |
| Tumor size (cm^3^) |  |  |  |
| ≤3 | 15 | 27 | 0.566 |
| 3-5 | 22 | 25 |  |
| >5 | 18 | 24 |  |
| Differentiation |  |  |  |
| Well/moderate | 18 | 37 | 0.049 |
| Poor/undifferentiation | 38 | 38 |  |
| Tumor invasion |  |  |  |
| T1/T2 | 13 | 23 | 0.402 |
| T3/T4 | 42 | 53 |  |
| TNM stage |  |  |  |
| I | 10 | 20 | 0.277 |
| II | 9 | 12 |  |
| III | 33 | 41 |  |
| IV | 3 | 3 |  |
| Lymph node metastasis (LNM) |  |  |  |
| Yes | 40 | 41 | 0.029 |
| No | 15 | 35 |  |
| No. of LNM |  |  |  |
| N0 | 15 | 35 | 0.126 |
| N1 (1-6) | 24 | 24 |  |
| N2 (7-15) | 14 | 13 |  |
| N3 (≥16) | 2 | 4 |  |
| Survival status |  |  |  |
| Dead | 37 | 29 | 0.001 |
| Alive | 18 | 47 |  |

**Supplementary Table 2.** *EHF* amplification in gastric cancer ― multivariable models assessing differentiation, Lymph node metastasis and survival status

| **Characteristics** | ***EHF* amplification [OR^†^ (95% CI)]** | ***P*** |
| --- | --- | --- |
| Differentiation^2^ | 1.458 (0.850-2.502) | 0.171 |
| Lymph node metastasis | 0.859 (0.516-1.430) | 0.558 |
| Survival status^4^ | 3.748 (1.525-9.214) | 0.004 |

**^†^**OR, odds ratio; CI, conﬁdence interval; ^1^Age (per 10 years); ^2^Differentiation (well or moderate; poor or no differentiation); ^3^TNM stage (I; II; III; IV); ^4^Survival status (Alive vs. Dead).

**Supplementary Table 3.** The sequences of siRNAs used in this study

| **si-RNAs** | **Sequences (5’-3’)** |
| --- | --- |
| si-EHF-309 (sense) | GCCAGUGGCAUGAAAUUCATT |
| si-EHF-309 (antisense) | UGAAUUUCAUGCCACUGGCTT |
| si-EHF-979 (sense) | CAGCCGAGCUAUGAGAUAUTT |
| si-EHF-979 (antisense) | AUAUCUCAUAGCUCGGCUGTT |
| si-HER2 -1299 (sense) | GCAGUUACCAGUGCCAAUATT |
| si-HER2-1299 (antisense) | UAUUGGCACUGGUAACUGCTT |
| si-NC (sense) | UUCUCCGAACGUGUCACGUTT |
| si-NC (antisense) | ACGUGACACGUUCGGAGAATT |

**Supplementary Table 4.** qRT-PCR primers used in this study

| **Genebank (ID)** | **Genes** | **Forward primer (5’-3’)** | **Reverse primer (5’-3’)** | **Product length (bp)** |
| --- | --- | --- | --- | --- |
| NM_[001199.3](http://www.ncbi.nlm.nih.gov/nuccore/NM_001199.3) | *BMP1* | GGGACGTGAAGTTCAGGATG | TTCTCCTCCCCTGAATACCC | 103 |
| NM_[001202.3](http://www.ncbi.nlm.nih.gov/nuccore/NM_001202.3) | *BMP4* | TGGCTGTCAAGAATCATGGA | ATCAAACTAGCATGGCTCGC | 135 |
| NM_[000245.2](http://www.ncbi.nlm.nih.gov/nuccore/NM_000245.2) | *c-Met* | CTCCAGCATTTTTACGGACC | GCTGCAAAGCTGTGGTAAACT | 121 |
| NM_012153.5 | *EHF* | TGATTCTGGAAGGAGGTGGT | ATGTCGAACTCTTGGAAAGGG | 238 |
| NM_[005228.3](http://www.ncbi.nlm.nih.gov/nuccore/NM_005228.3) | *EGFR* | GGGCTCTGGAGGAAAAGAAA | AAATTCCCAAGGACCACCTC | 127 |
| NM_[001005862.2](http://www.ncbi.nlm.nih.gov/nuccore/NM_001005862.2) | *HER2* | ATCAACTGCACCCACTCCTG | TGATGAGGATCCCAAAGACCAC | 145 |
| NM_[001005915.1](http://www.ncbi.nlm.nih.gov/nuccore/NM_001005915.1) | *HER3* | AGTCATGAGGGCGAACGAC | TCACACTCAGGCCATTCAGA | 119 |
| NM_[001042599.1](http://www.ncbi.nlm.nih.gov/nuccore/NM_001042599.1) | *HER4* | ACGGGATCTGAGACTTCCAA | TTATTCTCCGTTCCTGCACA | 127 |
| NM_004530.4 | *MMP2* | TTGCTGGAGACAAATTCTGG | AAGAAGTAGCTGTGACCGCC | 148 |
| NM_[002423.3](http://www.ncbi.nlm.nih.gov/nuccore/NM_002423.3) | *MMP7* | GAGCTACAGTGGGAACAGGC | GCATCTCCTTGAGTTTGGCT | 103 |
| NM_004994.2 | *MMP9* | GCACTGCAGGATGTCATAGG | ACGACGTCTTCCAGTACCGA | 128 |
| NM_004995.3 | *MMP14* | AGCCATATTGCTGTAGCCAG | GTTGTCTCCTGCTCCCCCT | 105 |
| R_003286.2 | *18S* | CGCCGCTAGAGGTGAAATTC | CTTTCGCTCTGGTCCGTCTT | 52 |

**Supplementary Table 5.** The primers used for luciferase reporter plasmid constructs in this study

| **Plasmids** | **Position** | **Forward primer (5’-3’)** | **Reverse primer (5’-3’)** | **Restriction sites** |
| --- | --- | --- | --- | --- |
| pGL3-HER2-Luc | -607/+11 | CGGGGTACCAAGTCCTTTCGATGTGACTGTC | CCGCTCGAGCTGGTTTCTCCGGTCCCAAT | Kpn1& Xho1 |
|  | -175/+11 | CGGGGTACCGGAGAAAGTGAAGCTGGGAG | CCGCTCGAGCTGGTTTCTCCGGTCCCAAT | Kpn1& Xho1 |
|  | -607/-175 | CGGGGTACCAAGTCCTTTCGATGTGACTGTC | CCGCTCGAGCCTCTCTTCGCGCAGGC | Kpn1& Xho1 |
| pGL3-HER3-Luc | -997/+440 | CGGGGTACCAGGTTGCATATCAATAGGGAGC | CCCAAGCTTGACTCCGCAGAGGGTGAAG | KpnI & HindIII |
| pGL3-HER4-Luc | -697/+306 | CGGGGTACCTTCCGTAAGCGGCCCTCC | CCCAAGCTTGGAAGTCTCAGATCCCGTGC | KpnI & HindIII |

**Supplementary Table 6.** qRT-PCR primers used for ChIP assay in this study

| **Genes** | **Position** | **Forward primer (5’-3’)** | **Reverse primer (5’-3’)** | **Product length (bp)** |
| --- | --- | --- | --- | --- |
| *HER2* | P1: -604/-484 | TCCTTTCGATGTGACTGTCTCC | TGTGTTTACCTTGTGGCTTCC | 121bp |
|  | P2: -274/-155 | TGCATTTAGGGATTCTCCGA | ACTCCCAGCTTCACTTTCTC | 120bp |
|  | P3: -147/-37 | CCCAGACTTGTTGGAATGCAG | ATTCTTATACTTCCTCAAGCAGCC | 111bp |
| *HER3* | P1: -203/-81 | TTCGAGTCTGGGAGAAACTGAG | TAGCCGGTTGGTTCACTTGG | 123bp |
|  | P2: -77/+43 | GAGTTGAGTGATTTGGTTAATGGG | GAGGTCGAGATTCCGAAAGC | 120bp |
| *HER4* | P1: -252/-151 | ACAAGTGTGAGGAAAGCTGAGAG | TGCACCTCCAGTCCAAATCG | 102bp |
|  | P2: 101/+6 | GAAACCTACCGCCTCTTTGC | CGCTCCCTCCTCACTTTCTC | 107bp |

**Supplementary Table 7**. Oligonucleotides of HER2 used in this study (bold and underline indicate the altered bases in the GGAA ETS core)

| **Oligonucleotides** | **Sense (5’-3’)** | **Antisense (5’-3’)** |
| --- | --- | --- |
| WT | GAGGGCTGCTTGAGGAAGTATAAGAATG | CATTCTTATACTTCCTCAAGCAGCCCTC |
| MT1 | GAGGGCTGCTTGAG**AG**AGTATAAGAATG | CATTCTTATACT**CT**CTCAAGCAGCCCTC |
| MT2 | GAGGGCTGCTTGAGGAAG**CT**TAAGAATG | CATTCTTA**AG**CTTCCTCAAGCAGCCCTC |
